# Changes in the composition and function of bacterial communities during vermicomposting may explain beneficial properties of vermicompost

**DOI:** 10.1038/s41598-019-46018-w

**Published:** 2019-07-04

**Authors:** Jorge Domínguez, Manuel Aira, Allison R. Kolbe, María Gómez-Brandón, Marcos Pérez-Losada

**Affiliations:** 1 Grupo de Ecoloxía Animal (GEA), Universidade de Vigo, E-36310 Vigo, Pontevedra Spain; 2 Computational Biology Institute, Milken Institute School of Public Health, George Washington University, Ashburn, VA 20147 USA; 3 CIBIO-InBIO, Centro de Investigação em Biodiversidade e Recursos Genéticos, Universidade do Porto, Campus Agrário de Vairão, 4485-661 Vairão, Portugal; 4 Department of Biostatistics and Bioinformatics, Milken Institute School of Public Health, George Washington University, Washington, DC 20052 USA

**Keywords:** Environmental microbiology, Metagenomics

## Abstract

Vermicomposting is the process by which organic waste is broken down through the synergistic actions of earthworms and microbial communities. Although vermicomposting has been shown to effectively reduce organic biomass and generate high-quality fertilizer for plants, little is known about the bacterial communities that are involved in this decomposition process. Since optimization of vermicomposting for commercial use necessitates additional knowledge of the underlying biological processes, this study sought to characterize the bacterial succession involved in the vermicomposting of Scotch broom (*Cytisus scoparius*), a leguminous shrub that has become invasive around the world with consequences for the dynamics and productivity of the ecosystems they occupy. Scotch broom was processed in a pilot-scale vermireactor for 91 days with the earthworm species *Eisenia andrei*. Samples were taken at the initiation of vermicomposting, and days 14, 42 and 91, representing both active and mature stages of vermicomposting. Significant changes (*P* < 0.0001) in the bacterial community composition (richness and evenness) were observed throughout the process. Increases in taxonomic diversity were accompanied by increases in functional diversity of the bacterial community, including metabolic capacity, streptomycin and salicylic acid synthesis, and nitrification. These results highlight the role of bacterial succession during the vermicomposting process and provide evidence of microbial functions that may explain the beneficial effects of vermicompost on soil and plants.

## Introduction

Vermicompost is a nutrient-rich organic amendment generated from organic waste through the combined action of earthworms and microorganisms^1–3^. Although earthworms are key players in the process of vermicomposting, microorganisms perform the actual decomposition of organic matter (OM). Earthworms indirectly stimulate microbial biomass and activity through the aeration and fragmentation of OM, increasing the available surface area for microbes and thus affecting the composition and structure of the microbial communities^4,5^. The contributions of earthworms to the process can be grouped into two phases: (i) an active phase characterized by the ingestion and processing of the organic wastes by earthworms and, (ii) a maturation-like phase in which microbes degrade the earthworm-processed materials^1^.

Vermicompost is a nutrient-rich, peat-like material characterized by high porosity, high water-holding capacity, and low C:N ratio^6^. When used as an amendment for soil or other plant growth media, vermicompost stimulates growth, seed germination and development, flowering, and fruit production of a variety of plant species^7,8^. These positive effects on plant growth may be driven by various factors, such as improved availability of air and water, presence of plant-growth regulating substances, and mitigation or suppression of plant diseases^7,9^.

The decaying OM during vermicomposting changes dramatically over time as a result of fluctuating rates of degradation and temporal changes in microbial community composition^4,5^. These changes in microbial community composition are an example of microbial succession as recently shown by Aira *et al*.^10^ with regard to the microbiome composition of ageing casts. During composting, early microbial colonizers are mainly heterotrophs, and succession is driven by the organic carbon derived from the initial substrate^11^. Although the microbial succession of composting has been previously described^12–14^, the microbial communities involved in the various stages of vermicomposting are less well known^15–17^. To date, most vermicomposting studies have primarily focused on determining the microbial community composition and microbial diversity in mature vermicompost^18–21^. A period of aging is necessary for the proper use of vermicompost to promote plant growth and suppress plant diseases^9^. However, the duration of the maturation phase is not constant and may vary depending on how efficiently the active phase is performed^5^. As such, a detailed evaluation of the microbial communities involved in the active phase of vermicomposting could help to broaden our understanding about the performance of the process and shed light onto the properties of the final product.

Vermicomposting has been shown to effectively compost a diverse variety of organic wastes, such as sewage sludge, food and animal wastes, and other industrial or agricultural wastes^1^. Therefore, vermicomposting has potential to convert plant biomass to high-quality organic fertilizer. The leguminous shrub Scotch broom (*Cytisus scoparius*), characterized by bright yellow, pea-like flowers, is native to the Mediterranean basin but has become invasive around the world^22^. Its success as an invasive plant can be attributed to its prolific production of seeds, which remain viable for long periods, symbiosis with nitrogen-fixing bacteria, as well as its growth habit of forming dense stands^23^. Forest depletion and the abandonment of land previously used for agricultural purposes may enable the spread and establishment of this invasive shrub, which may have profound consequences for the dynamics and productivity of the ecosystems they occupy^24^. As such, it is of utmost importance to find profitable options for sustainable utilization of this invasive shrub in order to avoid any negative environmental impacts.

Scotch broom is a symbiotic N-fixing species with high quantities of P, K, and Ca, making it potentially useful as a high-nutrient fertilizer. However, Scotch broom also contains high levels of polyphenols which can cause phytotoxicity^25^. Vermicomposting was previously shown to be successful in reducing the mass of Scotch broom and converting it in a nutrient-rich and stabilized peat-like vermicompost without phytotoxicity attributed to its polyphenol content^25^. The aim of the present study was to further characterize the bacterial communities that participate in the process of vermicomposting of Scotch broom by using high-throughput sequencing and metagenomics analyses to assess taxonomic and phylogenetic bacterial diversity in a pilot-scale vermireactor over a period of 91 days. These data were then used to describe bacterial succession during vermicomposting and infer metabolic functions of the vermicompost microbiome.

## Results

### Variation of earthworm and microbial biomass and microbial activity during vermicomposting of Scotch broom

**Figure 1:**
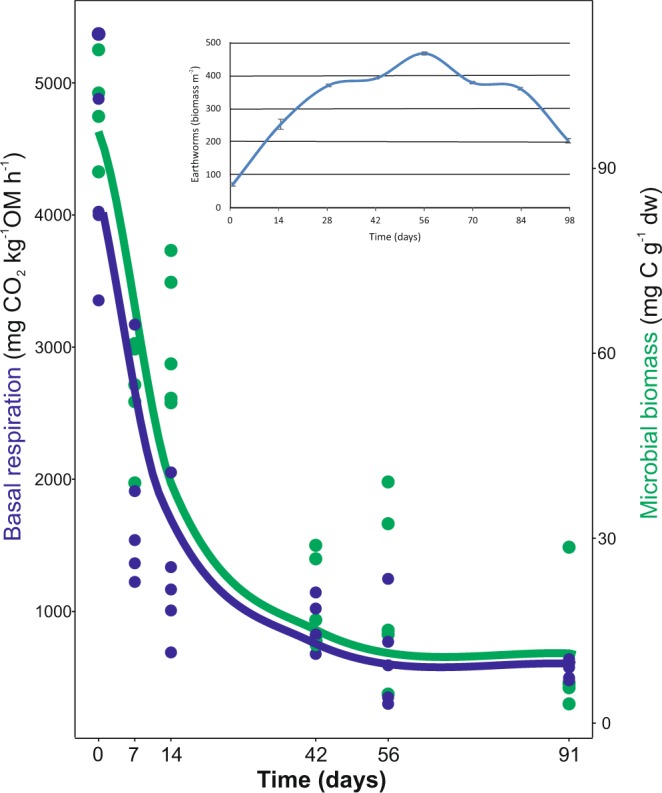
Changes in microbial biomass and microbial respiration during vermicomposting of the Scotch broom. Individual values (n = 5) are plotted for each time point, and the curve was plotted using the “loess” smoothing method in ggplot2^60^. The inset shows changes in earthworm biomass during the process. Earthworm biomass values are presented as means ± standard error (n = 5).

The total number of earthworms and earthworm biomass increased during the vermicomposting process until day 56, when the population reached its maximum density and biomass, with values of 1265 ± 20 individuals m^−2^ and 475.7 ± 1.9 g m^−2^, respectively (Fig. 1 inset). More in detail, the number of mature earthworms, juveniles, and cocoons increased significantly until day 56^25^. No additional Scotch broom was added to the vermireactor after the start of the experiment. Therefore, both earthworm numbers and biomass decreased until the end of the vermicomposting process on day 91. Similarly, microbial biomass and microbial activity (measured as basal respiration) decreased during vermicomposting, reaching their minimum values after 56 days and remaining constant until the end of the process (Fig. 1).

### Changes in bacterial community composition during vermicomposting of the Scotch broom

**Figure 2:**
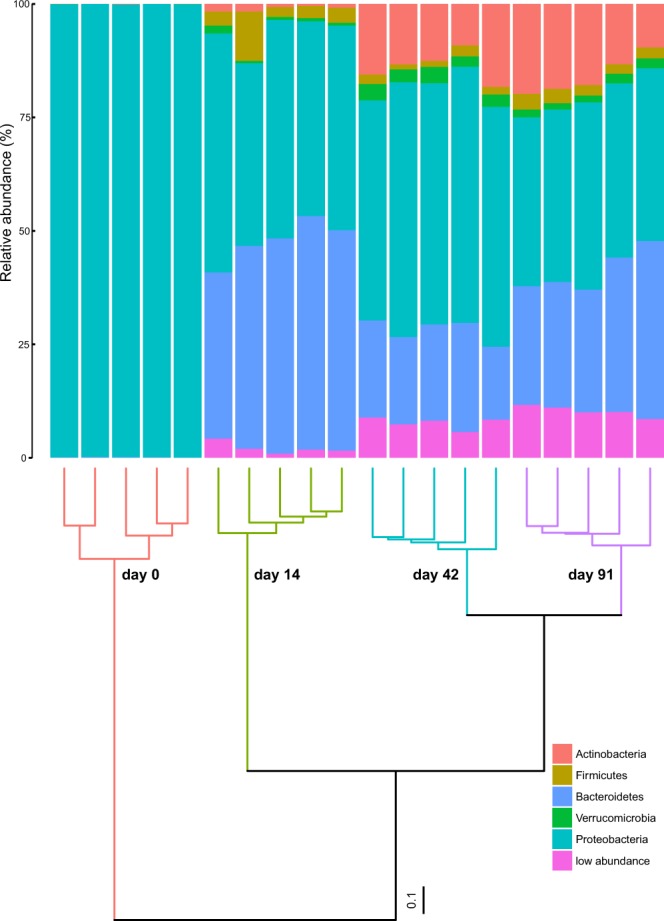
Changes in composition of the bacterial communities (phylum level) during vermicomposting of the Scotch broom. The dendrogram represents the dissimilarity of bacterial communities at ASV level (unweighted unifrac distances, Ward method). Bars represent the relative abundance of most abundant bacterial phyla. Low abundance bacterial phyla (<1%) were grouped together.

Bacterial community composition changed strongly during vermicomposting of the Scotch broom at the phylum (Fig. 2), class (Supplementary Figure S2) and amplicon sequence variant (ASV) levels (Supplementary Table S1). The fresh Scotch broom bacterial community was dominated by ASVs belonging to the phylum Proteobacteria (99.8% of sequences; Fig. 2; Table 1), but the bacterial composition during vermicomposting was split primarily between Proteobacteria, Bacteroidetes and Actinobacteria, with minor contributions of Firmicutes and Verrucomicrobia (Fig. 2; Table 1). Large changes in bacterial community composition were observed between 0 and 14 days, and between 14 and 42 days (Fig. 2). The bacterial community composition was relatively similar between the final time points of the experiment (days 42 and 91).

**Table 1:** Mean α-diversity indices, β-diversity indices, and mean relative abundances of dominant phyla and classes (>1%).

*Alpha diversity*		Day 0	Day 14	Day 42	Day 91	*F*_*3,12*_	*P(*>*F)*
Observed		30.0	405.6	619.2	554.2	41.94	<0.0001
Chao1		31.0	412.3	622.7	557.8	40.88	<0.0001
Shannon		1.1	4.7	5.6	5.1	384.95	<0.0001
Faith PD		5.7	41.5	71.1	64.7	148.82	<0.0001
***Beta diversity***		**Day 0**	**Day 14**	**Day 42**	**Day 91**	***F***_***3,12***_	**P-value**
Unifrac – unweighted	PCoA1	−0.56	0.09	0.23	0.23	1432.68	<0.001
	PCoA2	−0.06	0.39	−0.07	−0.25	317.84	<0.0001
Unifrac - weighted	PCoA1	0.59	−0.15	−0.20	−0.23	5966.55	<0.0001
	PCoA2	0.02	−0.31	0.05	0.22	143.96	<0.0001
Bray-Curtis	PCoA1	0.61	−0.01	−0.28	−0.32	2658.19	<0.001
	PCoA2	−0.18	0.53	−0.02	−0.31	240.74	<0.0001
Jaccard	PCoA1	0.60	−0.06	−0.24	−0.29	3985.57	<0.001
	PCoA2	−0.12	0.49	−0.04	−0.32	247.56	<0.0001
***Relative abundances – Phylum/Class***		**Day 0**	**Day 14**	**Day 42**	**Day 91**	***F***_***3,12***_	**P-value**
**Actinobacteria**		0.02	1.0	13.8	15.8	45.16	<0.0001
*Actinobacteria*		0.01	0.9	10.3	7.6	40.89	<0.0001
*Thermoleophilia*		0.003	0.06	2.7	6.7	53.5	<0.0001
**Bacteroidetes**		0.08	45.8	20.4	30.8	101.91	<0.0001
*Bacteroidia*		0	20.0	1.2	0.01	67.56	<0.0001
*Cytophagia*		0.04	7.5	3.1	2.8	22.27	<0.0001
*Flavobacteriia*		0.01	6.3	1.5	1.4	50.35	<0.0001
*Sphingobacteriia*		0.03	11.9	14.6	26.5	113.88	<0.0001
**Firmicutes**		0.02	4.4	1.7	2.7	4.95	0.0184
*Clostridia*		0	2.7	0.8	1.5	4.38	0.0265
**Proteobacteria**		99.9	45.8	53.4	38.6	443.90	<0.0001
*Alphaproteobacteria*		0.6	19.1	23.3	18.6	51.03	<0.0001
*Betaproteobacteria*		0.01	11.7	13.4	11.2	134.94	<0.0001
*Deltaproteobacteria*		0	0.7	4.3	2.6	50.46	<0.0001
*Gammaproteobacteria*		99.2	14.0	11.9	5.6	2406.09	<0.0001
**Verrucomicrobia**		0	0.8	3.0	1.8	72.36	<0.0001

Results from mixed-effects models are shown. Significance was determined using ANOVA. For each test, we report the relevant F statistic (*F*_*3,12*_) and significance (*P(*>*F))*.

### Changes in bacterial community diversity during vermicomposting of the Scotch broom

**Figure 3:**
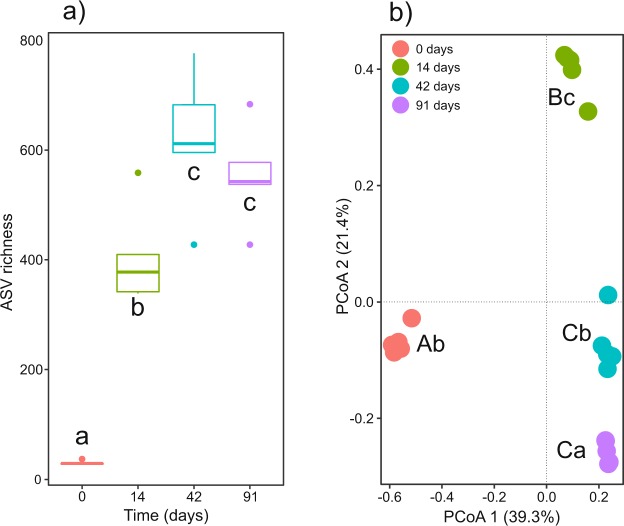
Changes in bacterial α-diversity and β-diversity during vermicomposting of the Scotch broom: (**a**) Observed ASV richness. Different letters indicate significant differences between the different stages of the vermicomposting process (Tukey HSD test, FDR corrected), (**b**) PCoA of unweighted UniFrac. Different capital and lower case letters indicate significant differences between the different stages of the vermicomposting process in PCoA 1 and PCoA 2 scores respectively (Tukey HSD test, FDR corrected).

Bacterial communities in the fresh dead Scotch broom (day 0) had a low α-diversity at both taxonomic (30 ± 2 ASVs) and phylogenetic level (Faith PD 5.67 ± 0.5) (Fig. 3). Between 0 and 42 days of vermicomposting there was a continuous and significant increase of α-diversity for ASV richness, Chao1 richness, Shannon diversity and Faith PD diversity (Fig. 3a; Table 1; Supplementary Figure S3). The increase in α-diversity is furthermore reflected in different patterns of phylogenetic and taxonomic β-diversity (Table 1; Supplementary Figure S4). At 91 days, there was a slight decrease of α- and β-diversity (Fig. 3, Supplementary Figure S3). However, bacterial communities were quite similar at both the taxonomic and phylogenetic level to those found after 42 days (Fig. 3b, Supplementary Figure S4).

### Core bacterial community within vermicomposting of Scotch broom

**Figure 4:**
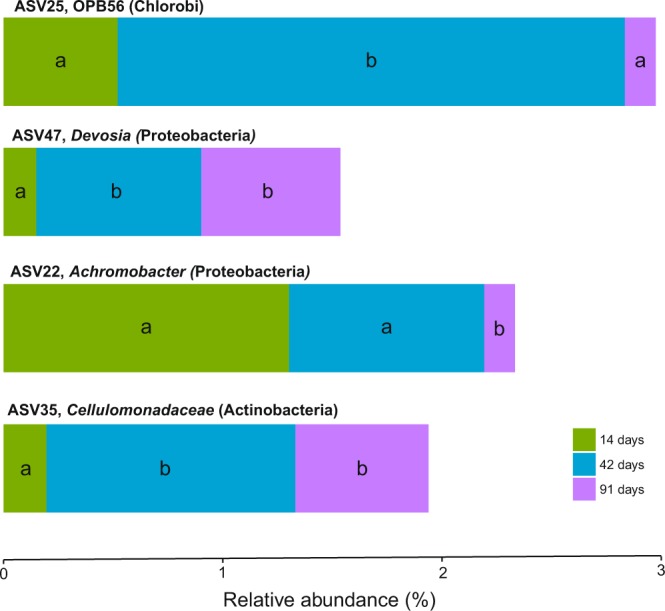
Relative abundance (%) of ASVs (phylum and genus or most inclusive taxonomy found) from the core microbiome of vermicomposting of the Scotch broom across days 14, 42 and 91. In all cases, relative abundance changed over time (ANOVA, p < 0.01). Different letters indicate significant differences between the different stages of the vermicomposting process (Tukey HSD test, FDR corrected).

The initial substrate (day 0) was not included in the core microbiome since this sample was not subjected to the action of earthworms. After removing the microbiome at day 0, the core microbiome of vermicomposting of Scotch broom comprised four bacteria that appeared in all the samples from days 14, 42 and 91 (0.07% of total ASVs and 2.2% of the sequences) (Fig. 4). These ASVs included one from the phylum Chlorobi (ASV25), two from Proteobacteria (*Devosia*: ASV47; *Achromobacter*: ASV22), and one Actinobacteria (*Cellulomonadaceae*: ASV35) (Fig. 4). The relative abundance of these four ASVs was significantly (*P* < 0.01) different between days 14, 42, and 91.

Additional shared ASVs were present when comparing time points. Only 3 ASVs were shared between days 0 and 14 (Supplementary Table S2), consistent with the large differences observed in bacterial community composition between these two time points. Between 14 and 42 days, 22 ASVs were shared; finally, between 42 and 91 days, 44 ASVs were shared (Supplementary Table S2). These results were consistent with the characterization of β-diversity, in which days 42 and 91 were the most closely related, and days 0 and 14 were distinct from other time points (Fig. 3b).

**Figure 5:**
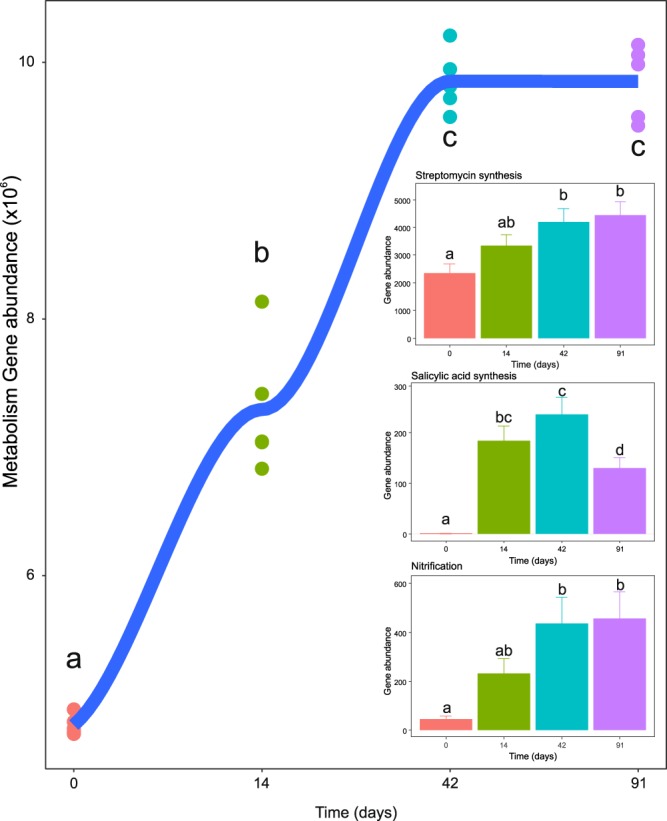
Changes in gene abundance of PICRUSt-predicted KEGG orthologies classified as “metabolism” in KEGG functional hierarchies during vermicomposting of the Scotch broom. Individual values (n = 5) are plotted for each time point, and the curve was plotted using the “loess” smoothing method in ggplot2. The insets show changes in gene abundance of all PICRUSt-predicted enzyme-level genes for synthesis of streptomycin and salicylic acid, and nitrification. Values are presented as means ± standard error (n = 5).

These shifts in bacterial taxa were related to significant changes in functional gene abundances, as predicted from 16S rRNA data analysis using PICRUSt. We detected significant predicted increases in genes classified only as “metabolism” in KEGG (Kyoto Encyclopedia of Genes and Genomes^26^) functional hierarchies (Fig. 5), and in genes related to nitrification and synthesis of streptomycin and salicylic acid (Fig. 5, inset). Genes involved in lignocellulose degradation varied in relative abundance during the course of vermicomposting. The relative abundances of microbial genes implicated in lignocellulose degradation during decomposition of the Scotch broom are summarized in Supplementary Table S3.

## Discussion

Although several studies have evaluated bacterial succession during composting, less is known about the effect of vermicomposting on bacterial community composition. This study provides a unique perspective on bacterial succession during vermicomposting of vegetal material and the Scotch broom in particular.

Previous work has described clear bacterial succession in thermophilic composting^12–14,27–29^, as well as changes in bacterial and fungal diversity^28,30^. Far fewer studies have evaluated temporal changes in bacterial composition during vermicomposting^15–17,29^. Lv *et al*.^29^ performed a comparative analysis of composting and vermicomposting of sewage sludge and cattle manure. They found an increase in bacterial diversity during vermicomposting, similar to that seen by Huang *et al*.^31^, who compared a 60-day-old vermicompost to a 60-day-old control without earthworms. Cai *et al*.^17^ also observed higher bacterial richness and diversity in vermicompost than in compost samples throughout the vermicomposting process of green waste. A main contribution of Cai *et al*.^17^ and our study is the incorporation of multiple time points during the active phase of vermicomposting, providing a more complete picture of bacterial succession. Vermicomposting of Scotch broom follows the normal pattern of an accelerated decomposition process in which there is a very rapid reduction in microbial biomass and activity, measured in this work as basal respiration (Fig. 1). This is consistent with previous findings of vermicomposting of vegetable wastes^31^. The evolution of these parameters indicated a good performance of the process, adequate for studying the succession of bacterial communities during vermicomposting.

Our data provide a strong example of bacterial succession driven by changes in the organic carbon source. During vermicomposting, the bacterial community composition can be classified in three large groups. The first group comprises the microbes present in the freshly cut Scotch broom, which has not been altered by earthworms (day 0). The second group is apparent on day 14, at which time point the community composition is comprised of bacteria that have recently passed through the intestines of earthworms and been excreted. Therefore, the increase in Bacteroidetes at day 14 (Table 1) is likely due to the gut associated processes, which is consistent with previous findings^17^. Later on, the quantity and quality of available nutrient supplies will gradually change as microbial succession progresses and this will be linked to the appearance of bacterial taxa specialized on metabolizing the remaining more recalcitrant substrates. In line with this, the final group, days 42 and 91, contains bacteria associated with the cast aging process. These two time points have a relatively similar bacterial community composition (Fig. 2, Supporting Information Figures S2 and S4), while differences between them can be attributed to the aging process of the casts.

There was also a significant increase in bacterial diversity, both taxonomic and phylogenetic, which peaked in the final succession group (day 42 and 91; Fig. 3 and Supporting Information Figure S3). Similarly, Cai *et al*.^17^ observed a higher bacterial richness and diversity in the final stages of the process, days 120 and 150, during vermicomposting of green waste. Gopal *et al*.^15^ observed a peak in bacterial diversity of coconut leaves mixed with cow dung slurry after 75 days of vermicomposting, followed by a decrease at the end of the trial on day 105. Such a decrease in the final stages of the process could be attributable to a reduction in the moisture level, since the vermicompost was heaped and left to air-dry without additional water from day 76 onwards^15^.

Although our results are generally similar to those in previous vermicomposting studies, the starting material is an important distinction of our study. Our study started with a homogenous substrate – Scotch broom – and therefore contained a less diverse microbiome than a heterogeneous mixture. The vermicompost produced in our study can be thought to represent the process of a single gut – that is, the starting material passed only through the earthworm gut. In contrast, many vermicomposting applications begin with animal manures or sewage sludge. In these cases, the starting material has already passed through the vertebrate gut (i.e. pig, cow, human) and therefore diversity may already be high. If vermicomposting starts with a highly diverse substrate, diversity could actually decrease during the process due to microbe depletion during worm gut passage. Previous studies have also emphasized the importance of the starting material for driving bacterial succession during vermicomposting^32^.

The bacterial succession during vermicomposting of Scotch broom is very clear, and the core microbiome (days 14, 42, and 91) includes only 4 ASVs. When including the initial time (day 0), there is no core microbiome. These results contrast with findings by Lv *et al*.^29^, who found increased diversity but similar overall bacterial composition between active and mature stages of vermicomposting. Specifically, within the core microbiome of Scotch broom, we detected the genus *Devosia* (order *Rhizobiales*), which is known to contribute to nitrogen fixation and can also confer plant protection through the release of plant growth promoting substances and antibiotics^17^. Members of the phylum Chlorobi and the family *Cellulomonodaceae*, as well as the genus *Achromobacter*, which are known to produce a large number of plant cell degrading enzymes^17^, were also present in the core microbiome of Scotch broom.

Variation in vermicomposting has been reported based on earthworm species, starting substrate, and other conditions^5,32^. Therefore, these results underscore the importance of further investigation into bacterial succession during vermicomposting with a variety of substrates and conditions. Furthermore, Lv, *et al*.^29^ collected samples at 20 days (active vermicomposting) and 80 days (mature composting). Our results indicate that significant changes happen within the first 14 days, so including additional time points may help to refine bacterial succession studies during vermicomposting.

In addition to an increase in genetic diversity during vermicomposting of Scotch broom, there was a clear increase in functional diversity (defined as PICRUSt-predicted KEGG orthologies). Functional diversity represents the metabolic capacity of the bacterial communities and can describe the way in which diverse microorganisms interact as a meta-organism to perform specific functions (e.g., the breakdown of lignocellulosic materials^33^). Community composition alterations at the phylum level include bacteria involved in the breakdown of the Scotch broom. For example, Actinobacteria are thought to be involved in decomposing cellulose and chitin during composting^28,34^, and Bacteroidetes have also been implicated in cellulose and hemicellulose degradation in soil^35^. However, in this study, genes involved in lignocellulosic degradation varied over the course of vermicomposting (Supporting Information Table S3). Analysis with PICRUSt found a general increase in metabolism genes (Fig. 5), which indicates that the initial bacterial community increased its metabolically activity with microbes serving diverse roles. In addition, specific metabolic processes, such as salicylic acid synthesis, streptomycin synthesis, and nitrification were also upregulated (Fig. 5 inset). These findings may support previous observations indicating improved plant performance when growing in vermicompost^7,9^. For example, salicylic acid has been shown to induce plant pathogen resistance mechanisms^36^, as well as to promote plant growth and development^37^. Streptomycin is produced by the soil microbe *Streptomyces griseus*, a member of Actinobacteria. The increase in streptomycin synthesis genes is paralleled by an increase in Actinobacteria during vermicomposting. Antibiotic production by beneficial soil microbes has been hypothesized as a mechanism for plant disease suppression in compost^38^. Streptomycin has been shown to control bacterial disease of some fruits, vegetables, and other crop species^39,40^; therefore, streptomycin production in vemicompost may confer disease resistance in some plants. Finally, increased nitrification is considered an indicator of mature compost^41^ so the increase in nitrification genes is expected during the vermicomposting procedure. In line with this, Huang, *et al*.^21^ also found that earthworms (*E. fetida*) facilitated the ammonia oxidization process by increasing the numbers (real time PCR-based) and diversity of ammonia-oxidizing bacteria and archaea during vermicomposting of fruit and vegetable wastes. Given that vermicompost has been shown to reduce impacts of plant pathogens, increase plant growth, and has other favorable benefits on plant performance, these alterations in functional diversity of the bacterial communities provide evidence for potential mechanisms by which such improvement is achieved.

In conclusion, our study provides evidence for succession of distinct bacterial communities during the active phase of vermicomposting, accompanied by significant changes in bacterial community functions. The taxonomic and functional microbial diversity generated during vermicomposting provides evidence for the beneficial properties of vermicompost as a fertilizer that stimulates plant growth, suppresses diseases, and protects plants against microbial pests.

## Methods

### Scotch broom (*Cytisus scoparius*)

Plants of the Scotch broom (*Cytisus scoparius* (L.) Link) were collected manually in a forest near the University of Vigo in spring, when the plants were flowering. Young branches were chopped (particle range size 3-6 cm) and flowers and leaves were left intact.

### Vermicomposting set-up and sampling design

Vermicomposting of fresh plant biomass (Scotch broom) was carried out in a rectangular metal pilot-scale vermireactor (4 m long × 1.5 m wide × 1 m high) housed in a greenhouse with no temperature control. The vermireactor set-up and sampling methods have been previously described^25,42,43^. Before adding the Scotch broom, the vermireactor contained a layer of vermicompost (12 cm height) as a bed for the earthworms (*Eisenia andrei)*. The initial earthworm population density in the vermireactor was 280 ± 9 individuals m^−2^, including 111 ± 10 mature earthworms m^−2^, 169 ± 7 immatures m^−2^ and 120 ± 3 cocoons m^−2^, with a mean biomass of 79.1 ± 5.2 g m^−2^. The fresh Scotch broom (120 kg fresh weight) was added to the bed in a 12 cm layer. The vermicompost bedding was separated from fresh Scotch broom by plastic mesh (5 mm mesh size). Use of the plastic mesh allows earthworm migration, prevents mixing of the processed Scotch broom and the vermicompost bedding and facilitates the sampling of Scotch broom during vermicomposting. The moisture content of this plant material was maintained at around 85% throughout the duration of the experiment by covering the vermireactor with a shade cloth^25^. The density and biomass of the earthworm population were determined periodically by collecting 10 samples (five from above and five from below the plastic mesh) of the material in the vermireactor every 14 days during the trial (91 days). The samples were collected with a core sampler (7.5 cm diameter and 12 cm height).

The physico-chemical characterization of the plant material from Scotch broom throughout the vermicomposting process is given in Domínguez, *et al*.^25^. For the characterization of the molecular and the microbial properties, in the present study the Scotch broom layer was divided into 5 sections, and two samples (10 g) were taken at random from each section at the beginning of the experiment (day 0) and after 14, 42 and 91 days of vermicomposting, as previously described^43^. The two samples from each quadrant were bulked and stored in plastic bags at -80 °C until analysis.

### Microbial biomass and activity

Microbial biomass C was analyzed by the chloroform fumigation–extraction method using a K_EC_ = 2.64^44^. Microbial activity was assessed as basal respiration, by measuring the rate of evolution of CO_2_ as modified by Aira, *et al*.^45^ for organic samples.

### DNA sequencing and bioinformatic analyses

DNA was extracted from 0.25 g (fresh weight) of Scotch broom using the MO-BIO PowerSoil® kit following the manufacturer’s protocols, as previously described^43^. DNA quality and quantity were determined using BioTek’s Take3™ Multi-Volume Plate. All laboratory procedures were performed under a laminar flow hood to prevent contamination of the samples with microorganisms from the surrounding environment. In order to describe the microbial succession during vermicomposting of Scotch broom, we focused on bacterial community composition by sequencing a fragment of the 16S rRNA gene covering the V4 region, by using a dual-index sequencing strategy, as described by Kozich, *et al*.^46^. In total, 20 DNA samples representing different sampling times (0, 14, 42 and 91 days) were sequenced using the Illumina MiSeq platform at the Genomics Core Facility of the Universitat Pompeu Fabra (Barcelona, Spain).

DADA2 (version 1.6) was used to infer the amplicon sequence variants (ASVs) present in each sample^47^, as previously described^48^. Exact sequence variants provide a more accurate and reproducible description of amplicon-sequenced communities than is possible with OTUs defined at a constant level (97% or other) of sequence similarity^49^. Bioinformatics processing largely followed the DADA2 pipeline tutorial (https://benjjneb.github.io/dada2/tutorial.html). Forward/reverse read pairs were trimmed and filtered, with forward reads truncated at 220 nt and reverse reads at 200 nt, no ambiguous bases allowed, and each read required to have less than two expected errors based on their quality scores. ASVs were independently inferred from the forward and reverse of each sample using the run-specific error rates, and then read pairs were merged. Chimeras were identified in each sample, and ASVs were removed if identified as chimeric in a sufficient fraction of the samples in which they were present. Taxonomic assignment was performed against the Silva v128 database using the implementation of the RDP naive Bayesian classifier available in the dada2 R package (min boot 80)^50,51^. A total of 869,932 sequences (mean: 43,496, SD: 16,377) passed all quality filters and were assigned to ASVs (5,404 and 3,346 before and after rarefaction respectively, without singletons and doubletons). We subsampled all samples to 17,373 sequences per sample to normalize the number of sequences. Rarefaction curves indicated that the sampling depth was optimal for Scotch broom and vermicompost samples (Supporting Information Figure S1).

Sequence data have been uploaded to the GenBank SRA database under accession number SRP120990.

### PICRUSt analysis

The functional composition of the metagenomes was predicted using the Phylogenetic Investigation of Communities by Reconstruction of Unobserved States software package (PICRUSt)^52^, as previously described^43^. Briefly, we first picked closed referenced operational taxonomic units (OTUs) (at 97%) against the 13_5 version of Greengenes database. The resulting OTU table was then normalized to account for known/predicted 16S copy number over which the functional composition of our metagenomes was predicted. The weighted nearest sequenced taxon index (NSTI) for our samples was 0.08 ± 0.04 (mean ± s.d.), which is acceptable as the samples are not from well described and/or sampled environments^52^. Predicted metagenomes were collapsed using the Kyoto Encyclopedia of Genes and Genomes (KEGG) Pathway metadata^26^.

### Statistical analysis

The samples were subsampled to the smallest sample size (17,373 sequences) to remove the effect of sample size bias on community composition, consistent with previous work^48^. An approximately maximum-likelihood phylogenetic tree was inferred using FastTree 2.1^53^. We defined the core microbiome of vermicomposting of the Scotch broom as that comprised of ASVs present in all the samples. Additionally, we evaluated the transitional core microbiomes as those comprised of ASVs present in all samples of 0 and 14 days, 14 and 42 days, and 42 and 91 days, as described in^54^.Taxonomic α-diversity was calculated as the number of observed ASVs, and diversity and richness were estimated with the Shannon and Chao1 indexes, respectively. Phylogenetic diversity was calculated as Faith’s phylogenetic diversity^55^. The effect of time (0, 14, 42, and 91 days) on both taxonomic and phylogenetic α-diversity of bacterial communities from the Scotch broom during vermicomposting was analyzed using mixed models in the ‘nlme’ R package^56^. Using the method described in^54^, time was the fixed factor and the effect of time nested in each sample was considered as a random factor to account for non-independence of samples due to repeated measures. The normality of residuals and homogeneity of variance across groups was checked for each variable. Tukey’s test was used for post-hoc comparisons, and Benjamini–Hochberg FDR was used as a multiple test correction method using the ‘multcomp’ package in R^57^. We used the same model to test for differences in the relative abundances of bacterial phyla and classes.

Taxonomic β-diversity at the ASV level was estimated as the difference in the composition of the bacterial taxonomic community between samples from different times during vermicomposting^43,48^. This was performed as previously described by coupling principal coordinate analysis (PCoA) with distance matrixes that take the abundance of ASVs into account (Bray–Curtis) or not (Jaccard)^43,48^. Phylogenetic β-diversity was also estimated by PCoA of weighted (considering abundance of ASVs) and unweighted unifrac matrix distances^58^ using the phyloseq library^59^. We utilized the mixed-models approach implemented in^43,48,54^ to analyze differences in β-diversity during vermicomposting, with PCoA scores as variables and time as fixed factor. As described above, the effect of time was nested in each sample as a random factor to account for non-independence of samples due to repeated measures. Tukey’s test was used for post-hoc comparisons and Benjamini–Hochberg FDR was used as multiple test correction method.

Mean values of relative abundance of gene contents (collapsed using KEGG Pathway metadata) from metagenomes of samples were separated with mixed models and post-hoc test as described above.

All figures were done with ggplot2 R package^60^. All analyses were performed with R version 3.1^61^.

## Supplementary information

Supplementary Information
