# Supplementary material for: Changes in the composition and function of bacterial communities during vermicomposting may explain beneficial properties of vermicompost
Source: Sci Rep. 2019 Jul 4;9:9657. doi: 10.1038/s41598-019-46018-w (PMC6609614; doi:10.1038/s41598-019-46018-w)
Supplement: Supplementary file 1 — Supplementary Information [file 41598_2019_46018_MOESM1_ESM.pdf]

Changes in the composition and function of bacterial communities during vermicomposting may explain beneficial properties of vermicompost

Jorge Domínguez[1], Manuel Aira[1], Allison R. Kolbe[2], María Gómez-Brandón[1*] & Marcos Pérez-Losada[2,3,4]

[1]Grupo de Ecoloxía Animal (GEA), Universidade de Vigo, E-36310, Spain.

[2]Computational Biology Institute, Milken Institute School of Public Health, George Washington University, Ashburn, VA 20147, USA.

[3]CIBIO-InBIO, Centro de Investigação em Biodiversidade e Recursos Genéticos, Universidade do Porto, Campus Agrário de Vairão, 4485-661 Vairão, Portugal.

[4]Department of Biostatistics and Bioinformatics, Milken Institute School of Public Health, George Washington University, Washington, DC 20052, USA

*Corresponding author at: Departamento de Ecoloxía e Bioloxía Animal, Universidade de Vigo, E-36310, Spain. Tel. +34 986 812593.

E-mail address: mariagomez@uvigo.es (M. Gómez-Brandón).

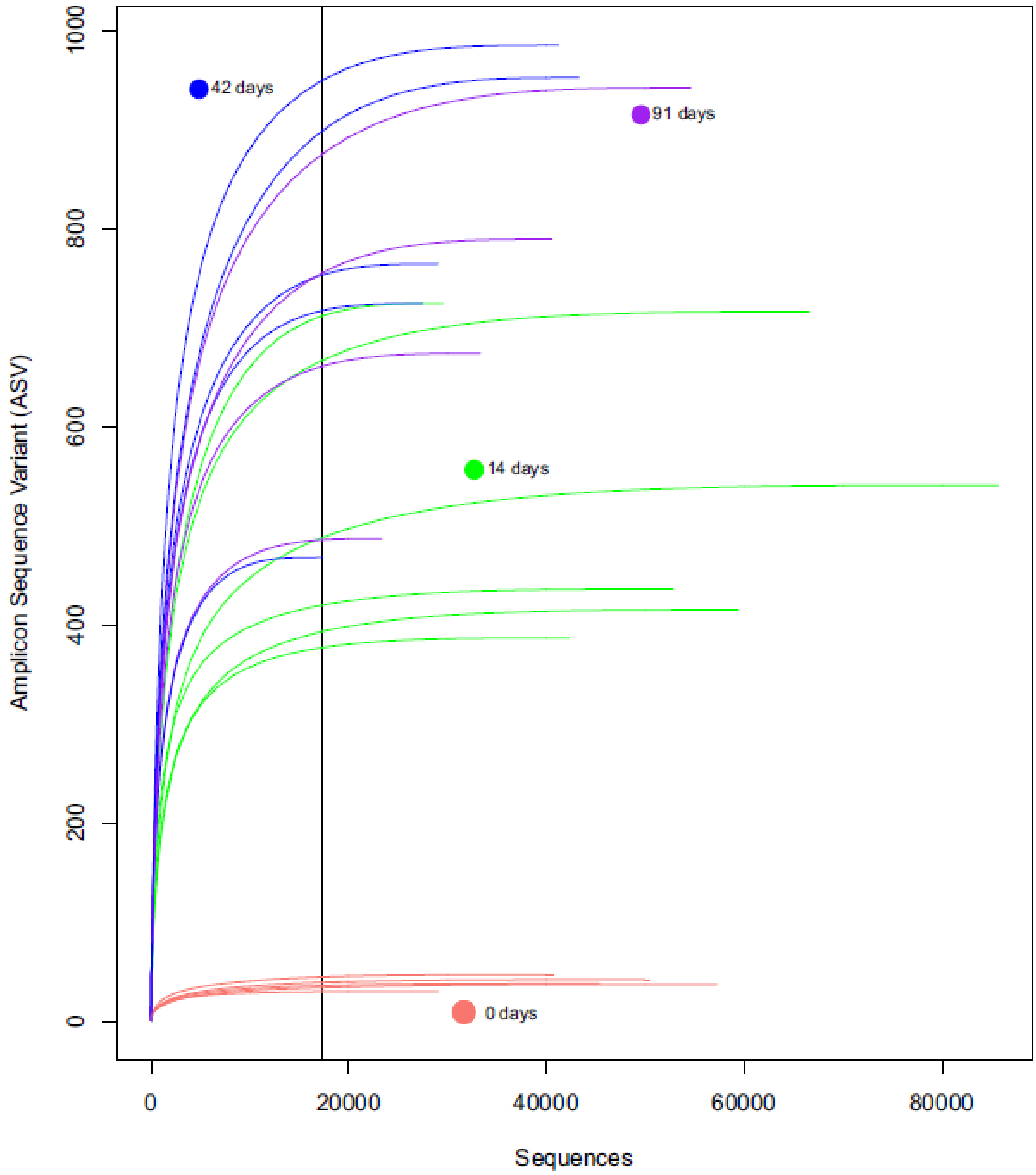

Fig. S1. Rarefaction curves indicating the number of amplicon sequence variants (ASVs) identified in bacterial communities found during vermicomposting of the Scotch broom: 0 (red), 14 (green), 42 (blue) and 91 days (purple).

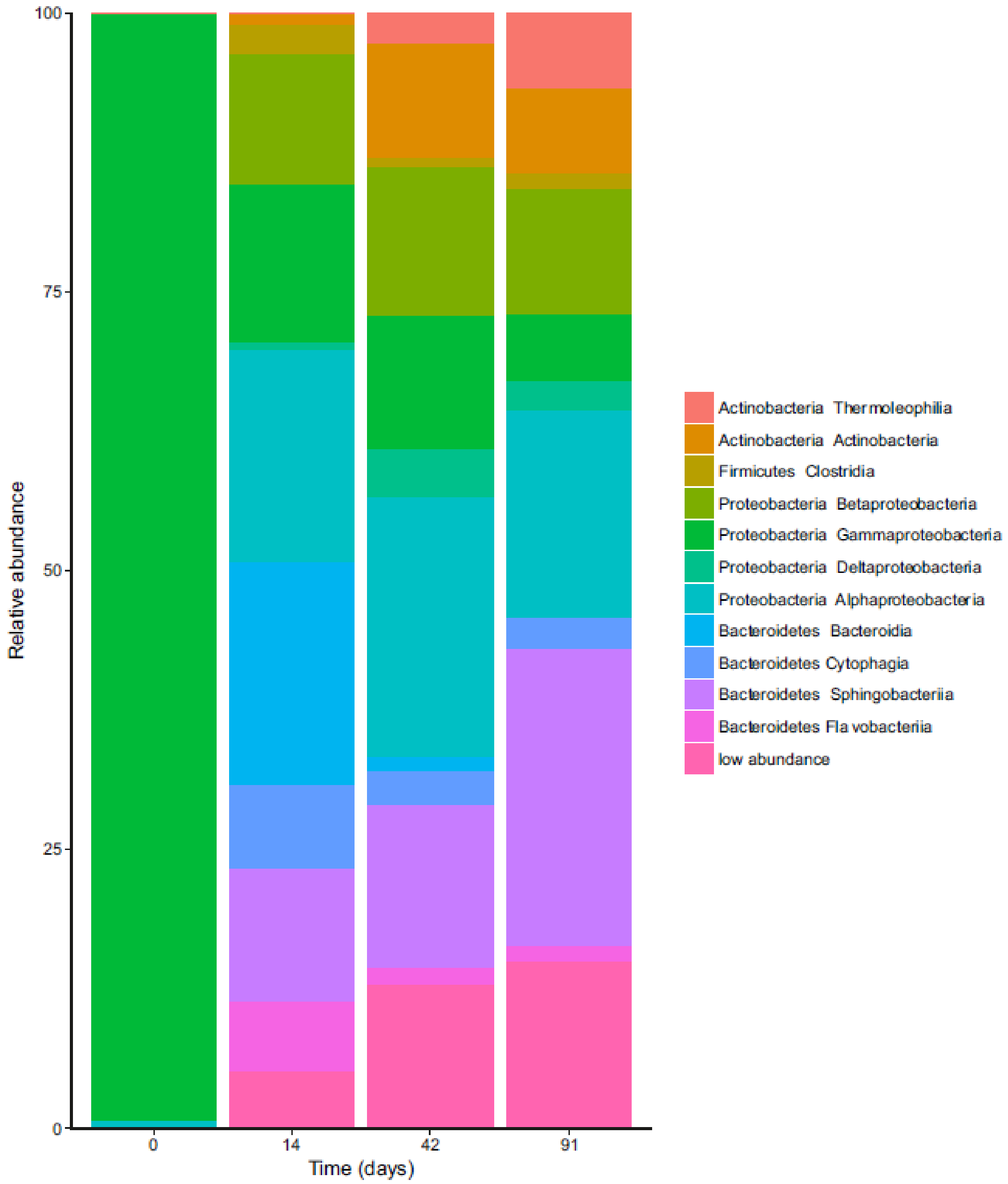

Fig. S2. Differences in relative abundance of main bacterial classes found during vermicomposting of Scotch broom (*Cytisus scoparius*). Low abundance bacterial classes (<1%) were grouped.

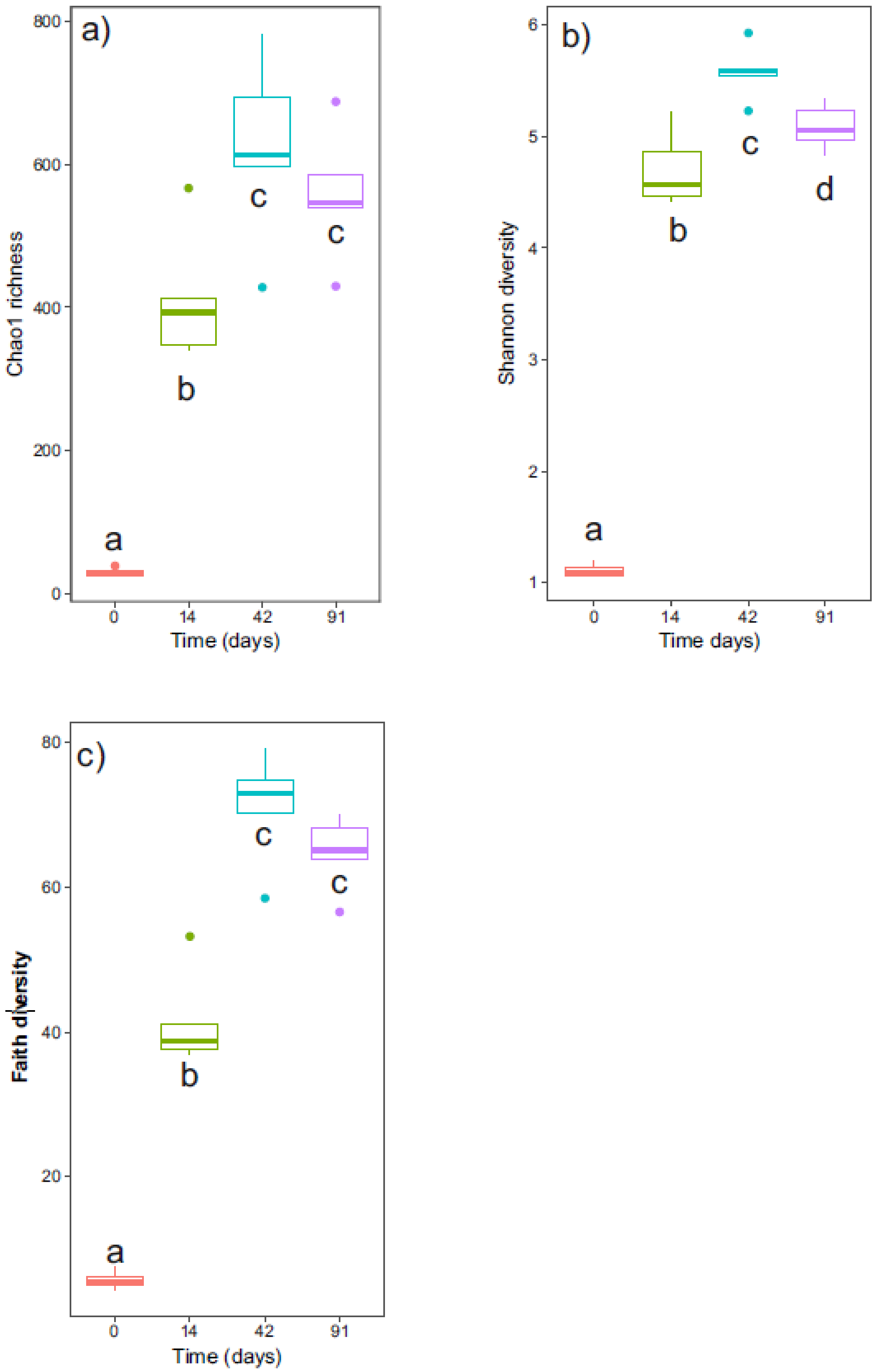

Fig. S3. Changes in bacterial α-diversity during vermicomposting of the Scotch broom: a) Taxonomic diversity (Shannon index), b) Estimated taxonomic richness (Chao 1), c) Phylogenetic diversity (Faith's PD). Different letters indicate significant differences between the different stages of the vermicomposting process (Tukey HSD test, FDR corrected)

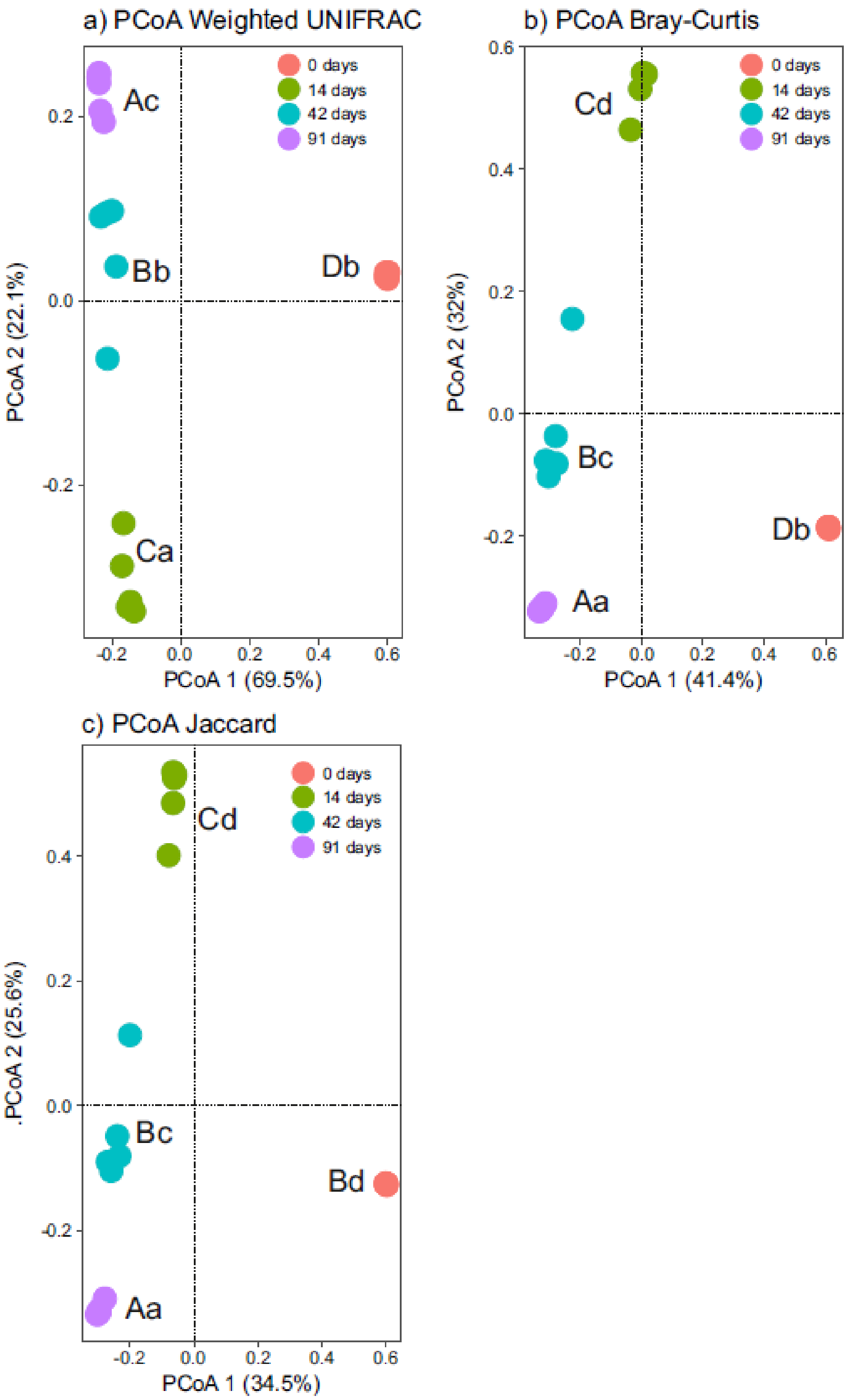

Fig. S4. Changes in bacterial β-diversity during vermicomposting of the Scotch broom: a) PCoA of weighted UniFrac, b) PCoA of Bray-Curtis, c) PCoA of Jaccard. Different capital and lower case letters indicate significant differences between the different stages of the vermicomposting process in PCoA 1 and PCoA 2 scores respectively (Tukey HSD test, FDR corrected).

Table S1. Taxonomic profile and relative abundance (Mean ± SE) of the bacterial core microbiome (ASVs) after 0, 14, 42 and 91 days of vermicomposting.

Time	Otu	Phylum	Class	Order	Family	Genus	Mean	SE
0	ASV307	Proteobacteria	Gammaproteobacteria	Pseudomonadales	Pseudomonadaceae	Pseudomonas	0.19	0.02
0	ASV56	Proteobacteria	Gammaproteobacteria	Pseudomonadales	Pseudomonadaceae	Pseudomonas	0.97	0.37
0	ASV2	Proteobacteria	Gammaproteobacteria	Enterobacteriales	Enterobacteriaceae	unclassified	14.01	0.43
0	ASV17	Proteobacteria	Gammaproteobacteria	Enterobacteriales	Enterobacteriaceae	unclassified	0.26	0.05
0	ASV202	Proteobacteria	Gammaproteobacteria	Enterobacteriales	Enterobacteriaceae	unclassified	0.22	0.02
0	ASV5	Proteobacteria	Gammaproteobacteria	Enterobacteriales	Enterobacteriaceae	unclassified	8.21	0.53
0	ASV1	Proteobacteria	Gammaproteobacteria	Enterobacteriales	Enterobacteriaceae	unclassified	67.41	0.83
0	ASV6	Proteobacteria	Gammaproteobacteria	Enterobacteriales	Enterobacteriaceae	unclassified	7.28	0.34
0	ASV269	Proteobacteria	Gammaproteobacteria	Xanthomonadales	Xanthomonadaceae	Stenotrophomonas	0.05	0.01
0	ASV517	Proteobacteria	Gammaproteobacteria	Xanthomonadales	Xanthomonadaceae	Luteibacter	0.08	0.02
0	ASV608	Proteobacteria	Alphaproteobacteria	Rhodospirillales	Acetobacteraceae	Acidiphilium	0.06	0.01
0	ASV30	Proteobacteria	Alphaproteobacteria	Caulobacterales	Caulobacteraceae	Brevundimonas	0.09	0.01
0	ASV301	Proteobacteria	Alphaproteobacteria	Sphingomonadales	Sphingomonadaceae	Sphingomonas	0.17	0.07
0	ASV7	Bacteroidetes	Cytophagia	Cytophagales	Cytophagaceae	Leadbetterella	0.03	0.01
14	ASV129	Planctomycetes	Planctomycetacia	Planctomycetales	Planctomycetaceae	Pirellula	0.31	0.15
14	ASV261	Tenericutes	Mollicutes	Mollicutes_RF9	unclassified	unclassified	0.13	0.03
14	ASV217	SR1_(Absconditabacteria)	unclassified	unclassified	unclassified	unclassified	0.18	0.05
14	ASV35	Actinobacteria	Actinobacteria	Micrococcales	Cellulomonadaceae	unclassified	0.20	0.05
14	ASV310	Firmicutes	Negativicutes	Selenomonadales	Veillonellaceae	Sporomusa	0.09	0.03
14	ASV588	Tenericutes	Mollicutes	unclassified	unclassified	unclassified	0.06	0.02
14	ASV397	Firmicutes	Bacilli	Lactobacillales	Streptococcaceae	Lactococcus	0.09	0.02
14	ASV119	Firmicutes	Bacilli	Lactobacillales	Leuconostocaceae	Weissella	0.27	0.05
14	ASV253	Firmicutes	Bacilli	Lactobacillales	Enterococcaceae	Enterococcus	0.14	0.06
14	ASV317	Acidobacteria	Blastocatellia	Blastocatellales	Blastocatellaceae_(Subgroup_4)	Blastocatella	0.05	0.02
14	ASV724	BRC1	unclassified	unclassified	unclassified	unclassified	0.03	0.01
14	ASV394	unclassified	unclassified	unclassified	unclassified	unclassified	0.09	0.02
14	ASV283	Verrucomicrobia	Verrucomicrobiae	Verrucomicrobiales	Verrucomicrobiaceae	Prosthecobacter	0.12	0.02
14	ASV270	Verrucomicrobia	Verrucomicrobiae	Verrucomicrobiales	Verrucomicrobiaceae	Verrucomicrobium	0.13	0.02
14	ASV299	Proteobacteria	Gammaproteobacteria	Pseudomonadales	Moraxellaceae	Perlucidibaca	0.09	0.05
14	ASV61	Proteobacteria	Betaproteobacteria	Rhodocyclales	Rhodocyclaceae	unclassified	0.61	0.31
14	ASV211	Proteobacteria	Betaproteobacteria	Rhodocyclales	Rhodocyclaceae	unclassified	0.16	0.09
14	ASV28	Proteobacteria	Betaproteobacteria	Burkholderiales	Burkholderiaceae	Burkholderia-Paraburkholderia	1.35	0.58
14	ASV257	Proteobacteria	Betaproteobacteria	Methylophilales	Methylophilaceae	Methylobacillus	0.14	0.04
14	ASV177	Proteobacteria	Betaproteobacteria	Burkholderiales	Alcaligenaceae	Parapusillimonas	0.13	0.03
14	ASV31	Proteobacteria	Betaproteobacteria	Burkholderiales	Alcaligenaceae	Achromobacter	0.73	0.12
14	ASV22	Proteobacteria	Betaproteobacteria	Burkholderiales	Alcaligenaceae	Achromobacter	1.30	0.30
14	ASV275	Proteobacteria	Betaproteobacteria	Neisseriales	Neisseriaceae	Microvirgula	0.13	0.06
14	ASV87	Proteobacteria	Betaproteobacteria	Burkholderiales	Comamonadaceae	unclassified	0.21	0.04
14	ASV76	Proteobacteria	Betaproteobacteria	Burkholderiales	Comamonadaceae	Xenophilus	0.39	0.06
14	ASV51	Proteobacteria	Betaproteobacteria	Burkholderiales	Comamonadaceae	unclassified	0.36	0.21
14	ASV36	Proteobacteria	Betaproteobacteria	Burkholderiales	Comamonadaceae	unclassified	1.19	0.31
14	ASV147	Proteobacteria	Betaproteobacteria	Burkholderiales	Comamonadaceae	unclassified	0.26	0.07
14	ASV54	Proteobacteria	Betaproteobacteria	Burkholderiales	Comamonadaceae	Hydrogenophaga	0.45	0.06

14	ASV206	Proteobacteria	Betaproteobacteria	Burkholderiales	Comamonadaceae	Comamonas	0.17	0.06
14	ASV91	Proteobacteria	Betaproteobacteria	Burkholderiales	Comamonadaceae	unclassified	0.44	0.13
14	ASV14	Proteobacteria	Betaproteobacteria	Burkholderiales	Comamonadaceae	unclassified	2.21	0.41
14	ASV209	Proteobacteria	unclassified	unclassified	unclassified	unclassified	0.16	0.06
14	ASV425	Proteobacteria	Gammaproteobacteria	Xanthomonadales	Xanthomonadales_Incertae_Sedis	Steroidobacter	0.05	0.01
14	ASV53	Proteobacteria	Gammaproteobacteria	Pseudomonadales	Pseudomonadaceae	Pseudomonas	0.59	0.15
14	ASV20	Proteobacteria	Gammaproteobacteria	Pseudomonadales	Pseudomonadaceae	Pseudomonas	1.92	0.52
14	ASV89	Proteobacteria	Gammaproteobacteria	Pseudomonadales	Pseudomonadaceae	Pseudomonas	0.30	0.09
14	ASV60	Proteobacteria	Gammaproteobacteria	Pseudomonadales	Pseudomonadaceae	Pseudomonas	0.77	0.12
14	ASV74	Proteobacteria	Gammaproteobacteria	Pseudomonadales	Pseudomonadaceae	Pseudomonas	0.48	0.29
14	ASV109	Proteobacteria	Gammaproteobacteria	Enterobacteriales	Enterobacteriaceae	unclassified	0.31	0.12
14	ASV17	Proteobacteria	Gammaproteobacteria	Enterobacteriales	Enterobacteriaceae	unclassified	1.72	0.29
14	ASV38	Proteobacteria	Gammaproteobacteria	Xanthomonadales	Xanthomonadaceae	Stenotrophomonas	0.99	0.40
14	ASV29	Proteobacteria	Gammaproteobacteria	Xanthomonadales	Xanthomonadaceae	Stenotrophomonas	0.66	0.20
14	ASV34	Proteobacteria	Gammaproteobacteria	Xanthomonadales	Xanthomonadaceae	Stenotrophomonas	1.14	0.24
14	ASV27	Proteobacteria	Gammaproteobacteria	Xanthomonadales	Xanthomonadaceae	Stenotrophomonas	1.57	0.56
14	ASV148	Proteobacteria	Gammaproteobacteria	Xanthomonadales	Xanthomonadaceae	Stenotrophomonas	0.28	0.07
14	ASV264	Proteobacteria	Gammaproteobacteria	Xanthomonadales	Xanthomonadaceae	Luteimonas	0.15	0.03
14	ASV233	Proteobacteria	Gammaproteobacteria	Xanthomonadales	Xanthomonadaceae	unclassified	0.07	0.04
14	ASV587	Proteobacteria	Deltaproteobacteria	Oligoflexales	Oligoflexaceae	unclassified	0.06	0.02
14	ASV414	Proteobacteria	Deltaproteobacteria	Bdellovibrionales	Bdellovibrionaceae	Bdellovibrio	0.08	0.02
14	ASV621	Proteobacteria	Deltaproteobacteria	Bdellovibrionales	Bdellovibrionaceae	Bdellovibrio	0.04	0.01
14	ASV501	Proteobacteria	Deltaproteobacteria	Myxococcales	BIrii41	unclassified	0.03	0.01
14	ASV44	Proteobacteria	Alphaproteobacteria	Rhodospirillales	Rhodospirillaceae	Azospirillum	1.01	0.42
14	ASV221	Proteobacteria	Alphaproteobacteria	Caulobacterales	Hyphomonadaceae	Hyphomonas	0.13	0.09
14	ASV127	Proteobacteria	Alphaproteobacteria	Caulobacterales	Caulobacteraceae	Brevundimonas	0.24	0.12
14	ASV26	Proteobacteria	Alphaproteobacteria	Caulobacterales	Caulobacteraceae	Brevundimonas	1.79	0.72
14	ASV32	Proteobacteria	Alphaproteobacteria	Caulobacterales	Caulobacteraceae	Brevundimonas	1.10	0.22
14	ASV30	Proteobacteria	Alphaproteobacteria	Caulobacterales	Caulobacteraceae	Brevundimonas	1.41	0.72
14	ASV8	Proteobacteria	Alphaproteobacteria	Caulobacterales	Caulobacteraceae	Asticcacaulis	3.44	0.80
14	ASV178	Proteobacteria	Alphaproteobacteria	Caulobacterales	Caulobacteraceae	Asticcacaulis	0.19	0.09
14	ASV201	Proteobacteria	Alphaproteobacteria	Rhizobiales	Rhizobiaceae	Rhizobium	0.10	0.04
14	ASV115	Proteobacteria	Alphaproteobacteria	Rhizobiales	Rhizobiaceae	Shinella	0.16	0.02
14	ASV328	Proteobacteria	Alphaproteobacteria	Rhizobiales	Brucellaceae	Ochrobactrum	0.11	0.03
14	ASV170	Proteobacteria	Alphaproteobacteria	Rhizobiales	Hyphomicrobiaceae	Devosia	0.13	0.05
14	ASV245	Proteobacteria	Alphaproteobacteria	Rhizobiales	Hyphomicrobiaceae	Devosia	0.04	0.01
14	ASV52	Proteobacteria	Alphaproteobacteria	Rhizobiales	Hyphomicrobiaceae	Devosia	0.44	0.20
14	ASV106	Proteobacteria	Alphaproteobacteria	Rhizobiales	Hyphomicrobiaceae	Devosia	0.29	0.05
14	ASV47	Proteobacteria	Alphaproteobacteria	Rhizobiales	Hyphomicrobiaceae	Devosia	0.15	0.07
14	ASV371	Proteobacteria	Alphaproteobacteria	Sphingomonadales	Sphingomonadaceae	unclassified	0.06	0.01
14	ASV429	Proteobacteria	Alphaproteobacteria	Sphingomonadales	Sphingomonadaceae	Sphingosinicella	0.05	0.02
14	ASV18	Proteobacteria	Alphaproteobacteria	Sphingomonadales	Sphingomonadaceae	Novosphingobium	1.93	0.84
14	ASV488	Proteobacteria	Alphaproteobacteria	Sphingomonadales	Sphingomonadaceae	Novosphingobium	0.04	0.01
14	ASV114	Proteobacteria	Alphaproteobacteria	Sphingomonadales	Sphingomonadaceae	Sphingopyxis	0.23	0.05
14	ASV403	Proteobacteria	Alphaproteobacteria	Sphingomonadales	Sphingomonadaceae	Sphingopyxis	0.07	0.02
14	ASV191	Proteobacteria	Alphaproteobacteria	Sphingomonadales	Sphingomonadaceae	Sphingopyxis	0.10	0.03

14	ASV258	Proteobacteria	Alphaproteobacteria	Rhodobacterales	Rhodobacteraceae	unclassified	0.13	0.04
14	ASV100	Proteobacteria	Alphaproteobacteria	Rhizobiales	Phyllobacteriaceae	Aquamicrobium	0.18	0.04
14	ASV263	Proteobacteria	Alphaproteobacteria	Rickettsiales	SM2D12	unclassified	0.13	0.04
14	ASV623	Proteobacteria	Alphaproteobacteria	Alphaproteobacteria_Incertae_Sedis	Unknown_Family	unclassified	0.04	0.02
14	ASV843	Proteobacteria	Alphaproteobacteria	Alphaproteobacteria_Incertae_Sedis	unclassified	unclassified	0.02	0.01
14	ASV564	Firmicutes	Clostridia	Clostridiales	Lachnospiraceae	unclassified	0.06	0.02
14	ASV181	Firmicutes	Clostridia	Clostridiales	Clostridiaceae_1	Clostridium_sensu_stricto_3	0.19	0.03
14	ASV604	Firmicutes	Clostridia	unclassified	unclassified	unclassified	0.06	0.04
14	ASV133	Firmicutes	unclassified	unclassified	unclassified	unclassified	0.28	0.09
14	ASV350	Firmicutes	Clostridia	Clostridiales	Ruminococcaceae	Intestinimonas	0.08	0.03
14	ASV15	Bacteroidetes	Sphingobacteriia	Sphingobacteriales	unclassified	unclassified	2.16	0.19
14	ASV186	Bacteroidetes	Cytophagia	Cytophagales	Cytophagaceae	Persicitalea	0.16	0.04
14	ASV324	Bacteroidetes	Cytophagia	Cytophagales	Cytophagaceae	Persicitalea	0.11	0.03
14	ASV167	Bacteroidetes	Cytophagia	Cytophagales	Cytophagaceae	Cytophaga	0.26	0.10
14	ASV244	Bacteroidetes	Flavobacteriia	Flavobacteriales	Flavobacteriaceae	Flavobacterium	0.14	0.05
14	ASV325	Bacteroidetes	Flavobacteriia	Flavobacteriales	Flavobacteriaceae	Flavobacterium	0.11	0.03
14	ASV482	Bacteroidetes	Flavobacteriia	Flavobacteriales	Flavobacteriaceae	Flavobacterium	0.05	0.02
14	ASV504	Bacteroidetes	Flavobacteriia	Flavobacteriales	Flavobacteriaceae	Flavobacterium	0.08	0.03
14	ASV78	Bacteroidetes	Flavobacteriia	Flavobacteriales	Flavobacteriaceae	Flavobacterium	0.50	0.21
14	ASV93	Bacteroidetes	Flavobacteriia	Flavobacteriales	Flavobacteriaceae	Myroides	0.40	0.14
14	ASV367	Bacteroidetes	Flavobacteriia	Flavobacteriales	Flavobacteriaceae	Flavobacterium	0.04	0.01
14	ASV65	Bacteroidetes	Bacteroidia	Bacteroidales	Porphyromonadaceae	Dysgonomonas	0.60	0.09
14	ASV311	Bacteroidetes	Bacteroidia	Bacteroidales	Porphyromonadaceae	Dysgonomonas	0.09	0.02
14	ASV9	Bacteroidetes	Bacteroidia	Bacteroidales	Porphyromonadaceae	Dysgonomonas	4.10	0.87
14	ASV10	Bacteroidetes	Bacteroidia	Bacteroidales	Porphyromonadaceae	Dysgonomonas	2.75	0.60
14	ASV4	Bacteroidetes	Bacteroidia	Bacteroidales	Porphyromonadaceae	Dysgonomonas	6.21	0.75
14	ASV288	Bacteroidetes	unclassified	unclassified	unclassified	unclassified	0.10	0.02
14	ASV42	Bacteroidetes	Sphingobacteriia	Sphingobacteriales	Sphingobacteriaceae	Sphingobacterium	0.93	0.29
14	ASV83	Bacteroidetes	Sphingobacteriia	Sphingobacteriales	Sphingobacteriaceae	Sphingobacterium	0.50	0.11
14	ASV225	Bacteroidetes	Sphingobacteriia	Sphingobacteriales	Sphingobacteriaceae	Nubsella	0.14	0.02
14	ASV63	Bacteroidetes	Sphingobacteriia	Sphingobacteriales	Sphingobacteriaceae	unclassified	0.66	0.18
14	ASV165	Bacteroidetes	Sphingobacteriia	Sphingobacteriales	Sphingobacteriaceae	Sphingobacterium	0.22	0.04
14	ASV116	Bacteroidetes	Sphingobacteriia	Sphingobacteriales	Sphingobacteriaceae	Sphingobacterium	0.40	0.17
14	ASV117	Bacteroidetes	Sphingobacteriia	Sphingobacteriales	Sphingobacteriaceae	unclassified	0.30	0.11
14	ASV352	Bacteroidetes	Sphingobacteriia	Sphingobacteriales	Sphingobacteriaceae	Sphingobacterium	0.11	0.02
14	ASV179	Bacteroidetes	Sphingobacteriia	Sphingobacteriales	Sphingobacteriaceae	Sphingobacterium	0.19	0.02
14	ASV364	Bacteroidetes	Sphingobacteriia	Sphingobacteriales	Sphingobacteriaceae	Sphingobacterium	0.09	0.04
14	ASV205	Bacteroidetes	Cytophagia	Cytophagales	unclassified	unclassified	0.16	0.04
14	ASV207	Bacteroidetes	Cytophagia	Cytophagales	Cytophagaceae	unclassified	0.17	0.04
14	ASV366	Bacteroidetes	Cytophagia	Cytophagales	Cytophagaceae	unclassified	0.09	0.03
14	ASV57	Bacteroidetes	Flavobacteriia	Flavobacteriales	NS9_marine_group	unclassified	0.73	0.42
14	ASV68	Bacteroidetes	Flavobacteriia	Flavobacteriales	Flavobacteriaceae	Moheibacter	0.41	0.15
14	ASV7	Bacteroidetes	Cytophagia	Cytophagales	Cytophagaceae	Leadbetterella	4.99	1.42
14	ASV11	Bacteroidetes	Bacteroidia	Bacteroidales	Bacteroidaceae	Bacteroides	2.97	0.24
14	ASV16	Bacteroidetes	Bacteroidia	Bacteroidales	Bacteroidaceae	Bacteroides	2.06	0.16
14	ASV25	Chlorobi	Chlorobia	Chlorobiales	OPB56	unclassified	0.52	0.30

14	ASV362	Bacteroidetes	Sphingobacteriia	Sphingobacteriales	Chitinophagaceae	Niabella	0.10	0.03
14	ASV90	Bacteroidetes	Sphingobacteriia	Sphingobacteriales	Chitinophagaceae	unclassified	0.47	0.16
14	ASV101	Bacteroidetes	Sphingobacteriia	Sphingobacteriales	Chitinophagaceae	unclassified	0.37	0.14
14	ASV852	Bacteroidetes	Sphingobacteriia	Sphingobacteriales	unclassified	unclassified	0.02	0.00
14	ASV341	Bacteroidetes	Sphingobacteriia	Sphingobacteriales	unclassified	unclassified	0.09	0.02
14	ASV652	Bacteroidetes	Flavobacteriia	Flavobacteriales	Flavobacteriaceae	Elizabethkingia	0.04	0.01
14	ASV86	Bacteroidetes	Flavobacteriia	Flavobacteriales	Flavobacteriaceae	Empedobacter	0.46	0.10
14	ASV33	Bacteroidetes	Flavobacteriia	Flavobacteriales	Flavobacteriaceae	Chryseobacterium	1.16	0.34
14	ASV203	Bacteroidetes	Sphingobacteriia	Sphingobacteriales	env.OPS_17	unclassified	0.16	0.04
14	ASV59	Bacteroidetes	Flavobacteriia	Flavobacteriales	Cryomorphaceae	Fluviicola	0.77	0.10
14	ASV99	Bacteroidetes	Flavobacteriia	Flavobacteriales	NS9_marine_group	unclassified	0.36	0.10
14	ASV23	Bacteroidetes	Sphingobacteriia	Sphingobacteriales	Chitinophagaceae	Taibaiella	1.63	0.49
14	ASV92	Bacteroidetes	Sphingobacteriia	Sphingobacteriales	Chitinophagaceae	Taibaiella	0.44	0.17
14	ASV406	Bacteroidetes	Sphingobacteriia	Sphingobacteriales	Chitinophagaceae	unclassified	0.08	0.02
14	ASV122	Bacteroidetes	Sphingobacteriia	Sphingobacteriales	Chitinophagaceae	Flavihumibacter	0.10	0.02
14	ASV615	Bacteroidetes	Sphingobacteriia	Sphingobacteriales	Chitinophagaceae	unclassified	0.03	0.02
42	ASV628	Planctomycetes	Planctomycetacia	Planctomycetales	Planctomycetaceae	Planctomyces	0.09	0.03
42	ASV946	unclassified	unclassified	unclassified	unclassified	unclassified	0.04	0.01
42	ASV24	Actinobacteria	Thermoleophilia	Gaiellales	Gaiellaceae	Gaiella	0.64	0.09
42	ASV64	Actinobacteria	Thermoleophilia	Solirubrobacterales	Elev-16S-1332	unclassified	0.28	0.05
42	ASV455	Actinobacteria	Thermoleophilia	Solirubrobacterales	Patulibacteraceae	Patulibacter	0.11	0.03
42	ASV214	Actinobacteria	Thermoleophilia	Solirubrobacterales	Solirubrobacteraceae	Solirubrobacter	0.14	0.02
42	ASV182	Actinobacteria	Actinobacteria	unclassified	unclassified	unclassified	0.36	0.06
42	ASV37	Actinobacteria	Actinobacteria	Kineosporiales	Kineosporiaceae	unclassified	1.46	0.25
42	ASV70	Actinobacteria	Actinobacteria	Micrococcales	Microbacteriaceae	Leucobacter	0.98	0.23
42	ASV88	Actinobacteria	Actinobacteria	Micrococcales	Demequinaceae	unclassified	0.45	0.05
42	ASV45	Actinobacteria	Actinobacteria	Micrococcales	Promicromonosporaceae	Cellulosimicrobium	0.94	0.17
42	ASV94	Actinobacteria	Actinobacteria	Micrococcales	Cellulomonadaceae	Cellulomonas	0.54	0.07
42	ASV35	Actinobacteria	Actinobacteria	Micrococcales	Cellulomonadaceae	unclassified	1.14	0.15
42	ASV77	Actinobacteria	Actinobacteria	Corynebacteriales	Mycobacteriaceae	Mycobacterium	0.46	0.06
42	ASV119	Firmicutes	Bacilli	Lactobacillales	Leuconostocaceae	Weissella	0.13	0.02
42	ASV407	Firmicutes	Bacilli	Bacillales	Paenibacillaceae	Paenibacillus	0.08	0.02
42	ASV317	Acidobacteria	Blastocatellia	Blastocatellales	Blastocatellaceae_(Subgroup_4)	Blastocatella	0.09	0.01
42	ASV62	Chloroflexi	KD4-96	unclassified	unclassified	unclassified	0.19	0.04
42	ASV95	Acidobacteria	Solibacteres	Solibacterales	Solibacteraceae_(Subgroup_3)	unclassified	0.32	0.05
42	ASV124	Acidobacteria	Solibacteres	Solibacterales	Solibacteraceae_(Subgroup_3)	Bryobacter	0.17	0.03
42	ASV704	Fibrobacteres	Fibrobacteria	Fibrobacterales	Fibrobacteraceae	unclassified	0.07	0.01
42	ASV118	Gemmatimonadetes	Gemmatimonadetes	Gemmatimonadales	Gemmatimonadaceae	unclassified	0.24	0.04
42	ASV499	Verrucomicrobia	Spartobacteria	Chthoniobacterales	Chthoniobacteraceae	Chthoniobacter	0.12	0.01
42	ASV149	Verrucomicrobia	Verrucomicrobiae	Verrucomicrobiales	Verrucomicrobiaceae	unclassified	0.51	0.10
42	ASV251	Verrucomicrobia	Verrucomicrobiae	Verrucomicrobiales	Verrucomicrobiaceae	unclassified	0.14	0.01
42	ASV177	Proteobacteria	Betaproteobacteria	Burkholderiales	Alcaligenaceae	Parapusillimonas	0.13	0.06
42	ASV31	Proteobacteria	Betaproteobacteria	Burkholderiales	Alcaligenaceae	Achromobacter	0.86	0.16
42	ASV22	Proteobacteria	Betaproteobacteria	Burkholderiales	Alcaligenaceae	Achromobacter	0.89	0.08
42	ASV87	Proteobacteria	Betaproteobacteria	Burkholderiales	Comamonadaceae	unclassified	0.40	0.14
42	ASV112	Proteobacteria	Betaproteobacteria	Burkholderiales	Comamonadaceae	Xenophilus	0.41	0.08

42	ASV51	Proteobacteria	Betaproteobacteria	Burkholderiales	Comamonadaceae	unclassified	0.92	0.15
42	ASV96	Proteobacteria	Betaproteobacteria	Burkholderiales	Comamonadaceae	unclassified	0.91	0.18
42	ASV58	Proteobacteria	Betaproteobacteria	Burkholderiales	Comamonadaceae	unclassified	0.96	0.23
42	ASV13	Proteobacteria	Betaproteobacteria	Burkholderiales	Comamonadaceae	unclassified	2.15	0.30
42	ASV73	Proteobacteria	Betaproteobacteria	Burkholderiales	Comamonadaceae	unclassified	0.49	0.09
42	ASV54	Proteobacteria	Betaproteobacteria	Burkholderiales	Comamonadaceae	Hydrogenophaga	0.73	0.15
42	ASV19	Proteobacteria	Betaproteobacteria	SC-I-84	unclassified	unclassified	0.61	0.08
42	ASV104	Proteobacteria	Betaproteobacteria	SC-I-84	unclassified	unclassified	0.53	0.11
42	ASV98	Proteobacteria	Betaproteobacteria	SC-I-84	unclassified	unclassified	0.21	0.04
42	ASV546	Proteobacteria	Betaproteobacteria	Nitrosomonadales	Nitrosomonadaceae	Nitrosomonas	0.05	0.01
42	ASV156	Proteobacteria	Gammaproteobacteria	Cellvibrionales	Cellvibrionaceae	Cellvibrio	0.20	0.01
42	ASV259	Proteobacteria	Gammaproteobacteria	Xanthomonadales	unclassified	unclassified	0.30	0.06
42	ASV168	Proteobacteria	Gammaproteobacteria	Xanthomonadales	Xanthomonadales_Incertae_Sedis	Steroidobacter	0.31	0.06
42	ASV89	Proteobacteria	Gammaproteobacteria	Pseudomonadales	Pseudomonadaceae	Pseudomonas	0.24	0.10
42	ASV74	Proteobacteria	Gammaproteobacteria	Pseudomonadales	Pseudomonadaceae	Pseudomonas	0.22	0.01
42	ASV471	Acidobacteria	Holophagae	Holophagales	Holophagaceae	Holophaga	0.10	0.02
42	ASV218	Proteobacteria	Gammaproteobacteria	Cellvibrionales	Halieaceae	unclassified	0.31	0.05
42	ASV17	Proteobacteria	Gammaproteobacteria	Enterobacteriales	Enterobacteriaceae	unclassified	0.46	0.07
42	ASV210	Proteobacteria	Gammaproteobacteria	Pseudomonadales	Moraxellaceae	unclassified	0.28	0.09
42	ASV29	Proteobacteria	Gammaproteobacteria	Xanthomonadales	Xanthomonadaceae	Stenotrophomonas	1.33	1.14
42	ASV66	Proteobacteria	Gammaproteobacteria	Xanthomonadales	Xanthomonadaceae	unclassified	0.85	0.20
42	ASV79	Proteobacteria	Gammaproteobacteria	Xanthomonadales	Xanthomonadaceae	Luteimonas	0.61	0.06
42	ASV40	Proteobacteria	Gammaproteobacteria	Xanthomonadales	Xanthomonadaceae	Rhodanobacter	0.57	0.10
42	ASV131	Proteobacteria	Gammaproteobacteria	Xanthomonadales	Xanthomonadaceae	Pseudoxanthomonas	0.49	0.10
42	ASV230	Proteobacteria	Gammaproteobacteria	Xanthomonadales	Xanthomonadaceae	Arenimonas	0.29	0.06
42	ASV154	Proteobacteria	Gammaproteobacteria	Xanthomonadales	Xanthomonadaceae	Thermomonas	0.48	0.15
42	ASV260	Proteobacteria	Gammaproteobacteria	Xanthomonadales	Xanthomonadaceae	Thermomonas	0.29	0.09
42	ASV1367	Proteobacteria	Gammaproteobacteria	unclassified	unclassified	unclassified	0.03	0.01
42	ASV768	Proteobacteria	Gammaproteobacteria	unclassified	unclassified	unclassified	0.08	0.02
42	ASV624	Proteobacteria	Gammaproteobacteria	HTA4	unclassified	unclassified	0.08	0.02
42	ASV456	Proteobacteria	Gammaproteobacteria	Legionellales	Coxiellaceae	Aquicella	0.16	0.06
42	ASV71	Proteobacteria	Deltaproteobacteria	Oligoflexales	Oligoflexaceae	Oligoflexus	0.92	0.10
42	ASV332	Proteobacteria	Deltaproteobacteria	Myxococcales	unclassified	unclassified	0.15	0.04
42	ASV497	Proteobacteria	Deltaproteobacteria	Myxococcales	BIrii41	unclassified	0.12	0.01
42	ASV50	Proteobacteria	Deltaproteobacteria	Myxococcales	Sandaracinaceae	unclassified	1.19	0.22
42	ASV80	Proteobacteria	Deltaproteobacteria	Myxococcales	Sandaracinaceae	unclassified	0.81	0.12
42	ASV384	Proteobacteria	Deltaproteobacteria	Myxococcales	Sandaracinaceae	unclassified	0.14	0.02
42	ASV159	Proteobacteria	Alphaproteobacteria	Rhodospirillales	Rhodospirillales_Incertae_Sedis	Reyranella	0.43	0.03
42	ASV492	Proteobacteria	Alphaproteobacteria	Rhodospirillales	Rhodospirillaceae	unclassified	0.10	0.02
42	ASV286	Proteobacteria	Alphaproteobacteria	Rhodospirillales	Rhodospirillales_Incertae_Sedis	Candidatus_Alysiosphaera	0.12	0.02
42	ASV127	Proteobacteria	Alphaproteobacteria	Caulobacterales	Caulobacteraceae	Brevundimonas	0.14	0.05
42	ASV228	Proteobacteria	Alphaproteobacteria	Caulobacterales	Caulobacteraceae	Caulobacter	0.24	0.07
42	ASV369	Proteobacteria	Alphaproteobacteria	Caulobacterales	Caulobacteraceae	Caulobacter	0.13	0.02
42	ASV349	Proteobacteria	Alphaproteobacteria	Caulobacterales	Caulobacteraceae	Caulobacter	0.19	0.02
42	ASV82	Proteobacteria	Alphaproteobacteria	Caulobacterales	Caulobacteraceae	Phenylobacterium	0.36	0.07
42	ASV97	Proteobacteria	Alphaproteobacteria	Caulobacterales	Caulobacteraceae	Asticcacaulis	0.57	0.12

42	ASV8	Proteobacteria	Alphaproteobacteria	Caulobacterales	Caulobacteraceae	Asticcacaulis	2.14	1.38
42	ASV294	Proteobacteria	Alphaproteobacteria	Caulobacterales	Caulobacteraceae	unclassified	0.26	0.08
42	ASV353	Proteobacteria	Alphaproteobacteria	Caulobacterales	Caulobacteraceae	unclassified	0.11	0.01
42	ASV201	Proteobacteria	Alphaproteobacteria	Rhizobiales	Rhizobiaceae	Rhizobium	0.18	0.10
42	ASV277	Proteobacteria	Alphaproteobacteria	Rhizobiales	Rhizobiaceae	Neorhizobium	0.12	0.02
42	ASV494	Proteobacteria	Alphaproteobacteria	Rhizobiales	Methylobacteriaceae	unclassified	0.13	0.03
42	ASV52	Proteobacteria	Alphaproteobacteria	Rhizobiales	Hyphomicrobiaceae	Devosia	0.61	0.13
42	ASV314	Proteobacteria	Alphaproteobacteria	Rhizobiales	Hyphomicrobiaceae	unclassified	0.17	0.05
42	ASV47	Proteobacteria	Alphaproteobacteria	Rhizobiales	Hyphomicrobiaceae	Devosia	0.75	0.08
42	ASV693	Proteobacteria	Alphaproteobacteria	Sphingomonadales	Sphingomonadaceae	unclassified	0.07	0.02
42	ASV197	Proteobacteria	Alphaproteobacteria	Sphingomonadales	Sphingomonadaceae	Sphingomonas	0.15	0.02
42	ASV383	Proteobacteria	Alphaproteobacteria	Sphingomonadales	unclassified	unclassified	0.20	0.05
42	ASV49	Proteobacteria	Alphaproteobacteria	Sphingomonadales	Sphingomonadaceae	Novosphingobium	1.01	0.13
42	ASV667	Proteobacteria	Alphaproteobacteria	Sphingomonadales	Sphingomonadaceae	unclassified	0.08	0.02
42	ASV39	Proteobacteria	Alphaproteobacteria	Sphingomonadales	Sphingomonadaceae	Novosphingobium	0.66	0.13
42	ASV132	Proteobacteria	Alphaproteobacteria	Sphingomonadales	Erythrobacteraceae	Altererythrobacter	0.26	0.08
42	ASV136	Proteobacteria	Alphaproteobacteria	Sphingomonadales	Erythrobacteraceae	Altererythrobacter	0.28	0.03
42	ASV110	Proteobacteria	Alphaproteobacteria	Rhodobacterales	Rhodobacteraceae	unclassified	0.30	0.06
42	ASV102	Proteobacteria	Alphaproteobacteria	Rhodobacterales	Rhodobacteraceae	unclassified	0.35	0.05
42	ASV55	Proteobacteria	Alphaproteobacteria	Rhodobacterales	Rhodobacteraceae	unclassified	0.54	0.09
42	ASV41	Proteobacteria	Alphaproteobacteria	unclassified	unclassified	unclassified	0.57	0.13
42	ASV619	Proteobacteria	Alphaproteobacteria	unclassified	unclassified	unclassified	0.13	0.02
42	ASV193	Proteobacteria	Alphaproteobacteria	unclassified	unclassified	unclassified	0.24	0.04
42	ASV1019	Proteobacteria	Alphaproteobacteria	Rickettsiales	Rickettsiaceae	Candidatus_Gigarickettsia	0.05	0.01
42	ASV274	Firmicutes	Clostridia	Clostridiales	Lachnospiraceae	Mobilitalea	0.10	0.04
42	ASV181	Firmicutes	Clostridia	Clostridiales	Clostridiaceae_1	Clostridium_sensu_stricto_3	0.09	0.02
42	ASV157	Bacteroidetes	Sphingobacteriia	Sphingobacteriales	Chitinophagaceae	Chitinophaga	0.30	0.06
42	ASV229	Bacteroidetes	Sphingobacteriia	Sphingobacteriales	Chitinophagaceae	Chitinophaga	0.28	0.02
42	ASV138	Bacteroidetes	Cytophagia	Cytophagales	Cytophagaceae	Persicitalea	0.11	0.03
42	ASV666	Bacteroidetes	Cytophagia	Cytophagales	Flammeovirgaceae	Candidatus_Amoebophilus	0.09	0.02
42	ASV585	Bacteroidetes	Cytophagia	Cytophagales	Cytophagaceae	Cytophaga	0.09	0.02
42	ASV126	Bacteroidetes	Cytophagia	Cytophagales	Cytophagaceae	unclassified	0.59	0.09
42	ASV361	Bacteroidetes	Cytophagia	Cytophagales	Cytophagaceae	Ohtaekwangia	0.18	0.03
42	ASV125	Bacteroidetes	Cytophagia	Cytophagales	Cytophagaceae	Chryseolinea	0.45	0.07
42	ASV420	Bacteroidetes	Cytophagia	Cytophagales	Cytophagaceae	Chryseolinea	0.15	0.03
42	ASV139	Bacteroidetes	Sphingobacteriia	Sphingobacteriales	Sphingobacteriaceae	Mucilaginibacter	0.38	0.06
42	ASV256	Bacteroidetes	Sphingobacteriia	Sphingobacteriales	Sphingobacteriaceae	unclassified	0.17	0.03
42	ASV117	Bacteroidetes	Sphingobacteriia	Sphingobacteriales	Sphingobacteriaceae	unclassified	0.12	0.03
42	ASV379	Bacteroidetes	Flavobacteriia	Flavobacteriales	Flavobacteriaceae	Moheibacter	0.13	0.02
42	ASV25	Chlorobi	Chlorobia	Chlorobiales	OPB56	unclassified	2.31	0.53
42	ASV631	Chlamydiae	Chlamydiae	Chlamydiales	unclassified	unclassified	0.08	0.01
42	ASV452	Bacteroidetes	Sphingobacteriia	Sphingobacteriales	Chitinophagaceae	Terrimonas	0.10	0.01
42	ASV348	Bacteroidetes	Sphingobacteriia	Sphingobacteriales	Chitinophagaceae	Ferruginibacter	0.15	0.05
42	ASV196	Bacteroidetes	Sphingobacteriia	Sphingobacteriales	Chitinophagaceae	Terrimonas	0.21	0.10
42	ASV3	Bacteroidetes	Sphingobacteriia	Sphingobacteriales	Chitinophagaceae	unclassified	2.22	0.39
42	ASV12	Bacteroidetes	Sphingobacteriia	Sphingobacteriales	Chitinophagaceae	unclassified	1.69	0.28

42	ASV313	Bacteroidetes	Sphingobacteriia	Sphingobacteriales	Chitinophagaceae	unclassified	0.20	0.03
42	ASV69	Bacteroidetes	Sphingobacteriia	Sphingobacteriales	Chitinophagaceae	Filimonas	0.93	0.13
42	ASV596	Bacteroidetes	Sphingobacteriia	Sphingobacteriales	unclassified	unclassified	0.12	0.04
42	ASV578	Bacteroidetes	Flavobacteriia	Flavobacteriales	Flavobacteriaceae	Chryseobacterium	0.09	0.01
42	ASV524	Bacteroidetes	Sphingobacteriia	Sphingobacteriales	Chitinophagaceae	Taibaiella	0.13	0.02
42	ASV84	Bacteroidetes	Sphingobacteriia	Sphingobacteriales	Chitinophagaceae	unclassified	0.49	0.09
42	ASV930	Bacteroidetes	Sphingobacteriia	Sphingobacteriales	Chitinophagaceae	Arachidicoccus	0.03	0.01
42	ASV67	Bacteroidetes	Sphingobacteriia	Sphingobacteriales	Chitinophagaceae	Parafilimonas	0.74	0.10
42	ASV21	Bacteroidetes	Sphingobacteriia	Sphingobacteriales	Chitinophagaceae	Parafilimonas	1.02	0.16
42	ASV122	Bacteroidetes	Sphingobacteriia	Sphingobacteriales	Chitinophagaceae	Flavihumibacter	0.42	0.09
91	ASV516	Planctomycetes	Planctomycetacia	Planctomycetales	Planctomycetaceae	Pirellula	0.10	0.02
91	ASV175	Planctomycetes	Planctomycetacia	Planctomycetales	Planctomycetaceae	unclassified	0.36	0.02
91	ASV743	Planctomycetes	Planctomycetacia	Planctomycetales	Planctomycetaceae	Planctomyces	0.06	0.01
91	ASV435	Planctomycetes	Planctomycetacia	Planctomycetales	Planctomycetaceae	Planctomyces	0.11	0.00
91	ASV823	Planctomycetes	Phycisphaerae	CPla-3_termite_group	unclassified	unclassified	0.04	0.01
91	ASV507	Planctomycetes	Phycisphaerae	Phycisphaerales	Phycisphaeraceae	SM1A02	0.10	0.02
91	ASV24	Actinobacteria	Thermoleophilia	Gaiellales	Gaiellaceae	Gaiella	2.33	0.28
91	ASV103	Actinobacteria	Thermoleophilia	Gaiellales	Gaiellaceae	Gaiella	0.65	0.08
91	ASV539	Actinobacteria	Thermoleophilia	Gaiellales	unclassified	unclassified	0.11	0.02
91	ASV344	Actinobacteria	Thermoleophilia	Gaiellales	Gaiellaceae	Gaiella	0.17	0.03
91	ASV150	Actinobacteria	Thermoleophilia	Solirubrobacterales	Patulibacteraceae	Patulibacter	0.16	0.01
91	ASV335	Actinobacteria	Thermoleophilia	Solirubrobacterales	Elev-16S-1332	unclassified	0.13	0.03
91	ASV64	Actinobacteria	Thermoleophilia	Solirubrobacterales	Elev-16S-1332	unclassified	0.81	0.08
91	ASV330	Actinobacteria	Thermoleophilia	Solirubrobacterales	Elev-16S-1332	unclassified	0.15	0.02
91	ASV113	Actinobacteria	Thermoleophilia	Solirubrobacterales	Elev-16S-1332	unclassified	0.57	0.04
91	ASV343	Actinobacteria	Thermoleophilia	Solirubrobacterales	unclassified	unclassified	0.14	0.02
91	ASV214	Actinobacteria	Thermoleophilia	Solirubrobacterales	Solirubrobacteraceae	Solirubrobacter	0.18	0.03
91	ASV37	Actinobacteria	Actinobacteria	Kineosporiales	Kineosporiaceae	unclassified	0.63	0.08
91	ASV45	Actinobacteria	Actinobacteria	Micrococcales	Promicromonosporaceae	Cellulosimicrobium	0.73	0.09
91	ASV35	Actinobacteria	Actinobacteria	Micrococcales	Cellulomonadaceae	unclassified	0.61	0.09
91	ASV811	Actinobacteria	Actinobacteria	Micrococcales	Brevibacteriaceae	Brevibacterium	0.06	0.01
91	ASV77	Actinobacteria	Actinobacteria	Corynebacteriales	Mycobacteriaceae	Mycobacterium	0.48	0.10
91	ASV120	Actinobacteria	Actinobacteria	Corynebacteriales	Mycobacteriaceae	Mycobacterium	0.38	0.08
91	ASV172	Actinobacteria	Actinobacteria	Propionibacteriales	Nocardioidaceae	unclassified	0.39	0.04
91	ASV192	Actinobacteria	Actinobacteria	Propionibacteriales	Nocardioidaceae	Nocardioides	0.32	0.05
91	ASV240	Actinobacteria	Acidimicrobiia	Acidimicrobiales	Acidimicrobiaceae	unclassified	0.28	0.01
91	ASV271	Actinobacteria	Acidimicrobiia	Acidimicrobiales	unclassified	unclassified	0.16	0.03
91	ASV174	Actinobacteria	Acidimicrobiia	Acidimicrobiales	unclassified	unclassified	0.34	0.05
91	ASV224	Firmicutes	Clostridia	Clostridiales	Peptostreptococcaceae	Romboutsia	0.24	0.03
91	ASV289	Firmicutes	Bacilli	Bacillales	unclassified	unclassified	0.16	0.03
91	ASV454	Firmicutes	Erysipelotrichia	Erysipelotrichales	Erysipelotrichaceae	Turicibacter	0.12	0.01
91	ASV709	Acidobacteria	Blastocatellia	Blastocatellales	Blastocatellaceae_(Subgroup_4)	unclassified	0.08	0.03
91	ASV531	Acidobacteria	Blastocatellia	Blastocatellales	Blastocatellaceae_(Subgroup_4)	unclassified	0.10	0.03
91	ASV107	Chloroflexi	KD4-96	unclassified	unclassified	unclassified	0.56	0.10
91	ASV62	Chloroflexi	KD4-96	unclassified	unclassified	unclassified	1.05	0.23
91	ASV268	Chloroflexi	KD4-96	unclassified	unclassified	unclassified	0.24	0.03

91	ASV95	Acidobacteria	Solibacteres	Solibacterales	Solibacteraceae_(Subgroup_3)	unclassified	0.51	0.06
91	ASV153	Acidobacteria	Solibacteres	Solibacterales	Solibacteraceae_(Subgroup_3)	unclassified	0.44	0.08
91	ASV124	Acidobacteria	Solibacteres	Solibacterales	Solibacteraceae_(Subgroup_3)	Bryobacter	0.36	0.02
91	ASV753	Spirochaetae	Spirochaetes	Spirochaetales	Leptospiraceae	Turneriella	0.05	0.01
91	ASV118	Gemmatimonadetes	Gemmatimonadetes	Gemmatimonadales	Gemmatimonadaceae	unclassified	0.36	0.08
91	ASV382	Verrucomicrobia	Spartobacteria	Chthoniobacterales	Chthoniobacteraceae	Chthoniobacter	0.11	0.01
91	ASV949	Verrucomicrobia	Spartobacteria	Chthoniobacterales	unclassified	unclassified	0.05	0.01
91	ASV541	Verrucomicrobia	Spartobacteria	Chthoniobacterales	Chthoniobacterales_Incertae_Sedis	Terrimicrobium	0.11	0.01
91	ASV315	Verrucomicrobia	Spartobacteria	Chthoniobacterales	Chthoniobacterales_Incertae_Sedis	Terrimicrobium	0.20	0.03
91	ASV251	Verrucomicrobia	Verrucomicrobiae	Verrucomicrobiales	Verrucomicrobiaceae	unclassified	0.14	0.02
91	ASV213	Nitrospirae	Nitrospira	Nitrospirales	Nitrospiraceae	Nitrospira	0.16	0.02
91	ASV204	Verrucomicrobia	OPB35_soil_group	unclassified	unclassified	unclassified	0.23	0.05
91	ASV342	Verrucomicrobia	OPB35_soil_group	unclassified	unclassified	unclassified	0.12	0.03
91	ASV600	Proteobacteria	Betaproteobacteria	Burkholderiales	Burkholderiaceae	Burkholderia-Paraburkholderia	0.08	0.01
91	ASV111	Proteobacteria	Betaproteobacteria	Rhodocyclales	Rhodocyclaceae	Uliginosibacterium	0.65	0.09
91	ASV22	Proteobacteria	Betaproteobacteria	Burkholderiales	Alcaligenaceae	Achromobacter	0.14	0.01
91	ASV108	Proteobacteria	Betaproteobacteria	Burkholderiales	Oxalobacteraceae	Massilia	0.68	0.08
91	ASV112	Proteobacteria	Betaproteobacteria	Burkholderiales	Comamonadaceae	Xenophilus	0.30	0.03
91	ASV161	Proteobacteria	Betaproteobacteria	Burkholderiales	Comamonadaceae	unclassified	0.33	0.03
91	ASV58	Proteobacteria	Betaproteobacteria	Burkholderiales	Comamonadaceae	unclassified	0.50	0.03
91	ASV13	Proteobacteria	Betaproteobacteria	Burkholderiales	Comamonadaceae	unclassified	2.28	0.10
91	ASV73	Proteobacteria	Betaproteobacteria	Burkholderiales	Comamonadaceae	unclassified	0.61	0.06
91	ASV19	Proteobacteria	Betaproteobacteria	SC-I-84	unclassified	unclassified	2.98	0.24
91	ASV104	Proteobacteria	Betaproteobacteria	SC-I-84	unclassified	unclassified	0.28	0.05
91	ASV98	Proteobacteria	Betaproteobacteria	SC-I-84	unclassified	unclassified	0.50	0.04
91	ASV156	Proteobacteria	Gammaproteobacteria	Cellvibrionales	Cellvibrionaceae	Cellvibrio	0.29	0.04
91	ASV169	Proteobacteria	Gammaproteobacteria	Cellvibrionales	Cellvibrionaceae	Simiduia	0.39	0.04
91	ASV287	Proteobacteria	Gammaproteobacteria	Xanthomonadales	unclassified	unclassified	0.22	0.02
91	ASV40	Proteobacteria	Gammaproteobacteria	Xanthomonadales	Xanthomonadaceae	Rhodanobacter	1.35	0.18
91	ASV265	Proteobacteria	Gammaproteobacteria	Xanthomonadales	Xanthomonadaceae	Dokdonella	0.24	0.03
91	ASV171	Proteobacteria	Gammaproteobacteria	Xanthomonadales	Xanthomonadaceae	unclassified	0.37	0.06
91	ASV305	Proteobacteria	Gammaproteobacteria	Legionellales	Coxiellaceae	Aquicella	0.22	0.05
91	ASV661	Proteobacteria	Gammaproteobacteria	Legionellales	Coxiellaceae	Aquicella	0.07	0.01
91	ASV529	Proteobacteria	Gammaproteobacteria	unclassified	unclassified	unclassified	0.09	0.01
91	ASV508	Acidobacteria	Subgroup_6	unclassified	unclassified	unclassified	0.10	0.01
91	ASV71	Proteobacteria	Deltaproteobacteria	Oligoflexales	Oligoflexaceae	Oligoflexus	0.21	0.02
91	ASV413	Proteobacteria	Deltaproteobacteria	Desulfurellales	Desulfurellaceae	H16	0.12	0.01
91	ASV771	Proteobacteria	Deltaproteobacteria	Bdellovibrionales	Bdellovibrionaceae	Bdellovibrio	0.06	0.01
91	ASV377	Proteobacteria	Deltaproteobacteria	Myxococcales	Polyangiaceae	Sorangium	0.11	0.02
91	ASV339	Proteobacteria	Deltaproteobacteria	Myxococcales	Sandaracinaceae	unclassified	0.14	0.01
91	ASV863	Proteobacteria	Deltaproteobacteria	Myxococcales	Sandaracinaceae	unclassified	0.05	0.01
91	ASV329	Proteobacteria	Deltaproteobacteria	Myxococcales	Sandaracinaceae	unclassified	0.13	0.01
91	ASV50	Proteobacteria	Deltaproteobacteria	Myxococcales	Sandaracinaceae	unclassified	0.54	0.09
91	ASV80	Proteobacteria	Deltaproteobacteria	Myxococcales	Sandaracinaceae	unclassified	0.21	0.04
91	ASV468	Proteobacteria	Alphaproteobacteria	Rhodospirillales	Acetobacteraceae	Acidicaldus	0.07	0.01
91	ASV291	Proteobacteria	Alphaproteobacteria	Rhodospirillales	Rhodospirillales_Incertae_Sedis	Reyranella	0.14	0.01

91	ASV399	Proteobacteria	Alphaproteobacteria	Rhodospirillales	Rhodospirillaceae	Dongia	0.08	0.01
91	ASV243	Proteobacteria	Alphaproteobacteria	Rhodospirillales	MSB-1E8	unclassified	0.22	0.05
91	ASV135	Proteobacteria	Alphaproteobacteria	Caulobacterales	Caulobacteraceae	Phenylobacterium	0.50	0.06
91	ASV82	Proteobacteria	Alphaproteobacteria	Caulobacterales	Caulobacteraceae	Phenylobacterium	0.32	0.04
91	ASV97	Proteobacteria	Alphaproteobacteria	Caulobacterales	Caulobacteraceae	Asticcacaulis	0.31	0.06
91	ASV267	Proteobacteria	Alphaproteobacteria	Caulobacterales	Caulobacteraceae	Asticcacaulis	0.24	0.03
91	ASV409	Proteobacteria	Alphaproteobacteria	Rhizobiales	unclassified	unclassified	0.17	0.03
91	ASV393	Proteobacteria	Alphaproteobacteria	Rhizobiales	Rhizobiaceae	Ensifer	0.13	0.02
91	ASV142	Proteobacteria	Alphaproteobacteria	Rhizobiales	unclassified	unclassified	0.47	0.06
91	ASV72	Proteobacteria	Alphaproteobacteria	Rhizobiales	unclassified	unclassified	0.77	0.10
91	ASV562	Proteobacteria	Alphaproteobacteria	Rhizobiales	Hyphomicrobiaceae	unclassified	0.11	0.02
91	ASV47	Proteobacteria	Alphaproteobacteria	Rhizobiales	Hyphomicrobiaceae	Devosia	0.63	0.09
91	ASV577	Proteobacteria	Alphaproteobacteria	Rhizobiales	Rhizobiaceae	Kaistia	0.08	0.02
91	ASV141	Proteobacteria	Alphaproteobacteria	Rhizobiales	Roseiarcaceae	Roseiarcus	0.47	0.06
91	ASV197	Proteobacteria	Alphaproteobacteria	Sphingomonadales	Sphingomonadaceae	Sphingomonas	0.23	0.05
91	ASV292	Proteobacteria	Alphaproteobacteria	Sphingomonadales	Sphingomonadaceae	Sphingomonas	0.21	0.03
91	ASV183	Proteobacteria	Alphaproteobacteria	Sphingomonadales	Sphingomonadaceae	unclassified	0.36	0.05
91	ASV49	Proteobacteria	Alphaproteobacteria	Sphingomonadales	Sphingomonadaceae	Novosphingobium	0.58	0.05
91	ASV252	Proteobacteria	Alphaproteobacteria	Sphingomonadales	Sphingomonadaceae	Sphingobium	0.23	0.02
91	ASV304	Proteobacteria	Alphaproteobacteria	Sphingomonadales	Sphingomonadaceae	Novosphingobium	0.10	0.03
91	ASV39	Proteobacteria	Alphaproteobacteria	Sphingomonadales	Sphingomonadaceae	Novosphingobium	1.00	0.10
91	ASV132	Proteobacteria	Alphaproteobacteria	Sphingomonadales	Erythrobacteraceae	Altererythrobacter	0.35	0.06
91	ASV427	Proteobacteria	Alphaproteobacteria	Rhodobacterales	Rhodobacteraceae	unclassified	0.10	0.02
91	ASV144	Proteobacteria	Alphaproteobacteria	Rhodobacterales	Rhodobacteraceae	unclassified	0.32	0.03
91	ASV110	Proteobacteria	Alphaproteobacteria	Rhodobacterales	Rhodobacteraceae	unclassified	0.38	0.07
91	ASV102	Proteobacteria	Alphaproteobacteria	Rhodobacterales	Rhodobacteraceae	unclassified	0.34	0.10
91	ASV55	Proteobacteria	Alphaproteobacteria	Rhodobacterales	Rhodobacteraceae	unclassified	0.84	0.22
91	ASV41	Proteobacteria	Alphaproteobacteria	unclassified	unclassified	unclassified	1.13	0.20
91	ASV365	Proteobacteria	Alphaproteobacteria	Caulobacterales	Hyphomonadaceae	Hirschia	0.14	0.03
91	ASV193	Proteobacteria	Alphaproteobacteria	unclassified	unclassified	unclassified	0.13	0.04
91	ASV274	Firmicutes	Clostridia	Clostridiales	Lachnospiraceae	Mobilitalea	0.08	0.01
91	ASV336	Firmicutes	Clostridia	Clostridiales	Clostridiaceae_1	Clostridium_sensu_stricto_1	0.13	0.02
91	ASV385	Firmicutes	Clostridia	Clostridiales	Clostridiaceae_1	Clostridium_sensu_stricto_1	0.18	0.02
91	ASV548	Bacteroidetes	Cytophagia	Cytophagales	Cytophagaceae	unclassified	0.10	0.02
91	ASV498	Bacteroidetes	Cytophagia	Cytophagales	Cytophagaceae	Cytophaga	0.11	0.02
91	ASV48	Bacteroidetes	Cytophagia	Cytophagales	Cytophagaceae	Cytophaga	1.43	0.16
91	ASV134	Bacteroidetes	Cytophagia	Cytophagales	Cytophagaceae	unclassified	0.27	0.04
91	ASV254	Bacteroidetes	Cytophagia	Cytophagales	Cytophagaceae	unclassified	0.22	0.04
91	ASV654	Bacteroidetes	Cytophagia	Cytophagales	Cytophagaceae	Chryseolinea	0.07	0.03
91	ASV701	Bacteroidetes	Cytophagia	Cytophagales	Cytophagaceae	Cytophaga	0.06	0.01
91	ASV458	Bacteroidetes	Flavobacteriia	Flavobacteriales	Flavobacteriaceae	Flavobacterium	0.11	0.02
91	ASV184	Bacteroidetes	Flavobacteriia	Flavobacteriales	Flavobacteriaceae	Flavobacterium	0.28	0.06
91	ASV140	Bacteroidetes	Flavobacteriia	Flavobacteriales	Flavobacteriaceae	Flavobacterium	0.51	0.05
91	ASV465	Bacteroidetes	Flavobacteriia	Flavobacteriales	Flavobacteriaceae	Flavobacterium	0.13	0.03
91	ASV316	Bacteroidetes	Sphingobacteriia	Sphingobacteriales	Sphingobacteriaceae	unclassified	0.16	0.03
91	ASV400	Bacteroidetes	Sphingobacteriia	Sphingobacteriales	Sphingobacteriaceae	Mucilaginibacter	0.12	0.03

91	ASV25	Chlorobi	Chlorobia	Chlorobiales	OPB56	unclassified	0.14	0.04
91	ASV583	Chlamydiae	Chlamydiae	Chlamydiales	unclassified	unclassified	0.12	0.02
91	ASV223	Chlamydiae	Chlamydiae	Chlamydiales	unclassified	unclassified	0.23	0.03
91	ASV534	Chlamydiae	Chlamydiae	Chlamydiales	unclassified	unclassified	0.09	0.02
91	ASV448	Chlamydiae	Chlamydiae	Chlamydiales	unclassified	unclassified	0.12	0.02
91	ASV688	Chlamydiae	Chlamydiae	Chlamydiales	Parachlamydiaceae	unclassified	0.07	0.01
91	ASV461	Chlamydiae	Chlamydiae	Chlamydiales	cvE6	unclassified	0.12	0.02
91	ASV222	Bacteroidetes	Sphingobacteriia	Sphingobacteriales	Chitinophagaceae	unclassified	0.29	0.05
91	ASV3	Bacteroidetes	Sphingobacteriia	Sphingobacteriales	Chitinophagaceae	unclassified	14.45	1.24
91	ASV12	Bacteroidetes	Sphingobacteriia	Sphingobacteriales	Chitinophagaceae	unclassified	3.07	0.26
91	ASV180	Bacteroidetes	Sphingobacteriia	Sphingobacteriales	Chitinophagaceae	unclassified	0.35	0.10
91	ASV69	Bacteroidetes	Sphingobacteriia	Sphingobacteriales	Chitinophagaceae	Filimonas	0.24	0.05
91	ASV241	Bacteroidetes	Sphingobacteriia	Sphingobacteriales	Chitinophagaceae	Taibaiella	0.26	0.02
91	ASV281	Bacteroidetes	Sphingobacteriia	Sphingobacteriales	Chitinophagaceae	unclassified	0.22	0.02
91	ASV75	Bacteroidetes	Sphingobacteriia	Sphingobacteriales	Chitinophagaceae	unclassified	0.85	0.15
91	ASV173	Bacteroidetes	Sphingobacteriia	Sphingobacteriales	Chitinophagaceae	unclassified	0.33	0.04
91	ASV625	Bacteroidetes	Sphingobacteriia	Sphingobacteriales	Chitinophagaceae	unclassified	0.08	0.01
91	ASV576	Bacteroidetes	Sphingobacteriia	Sphingobacteriales	Chitinophagaceae	unclassified	0.08	0.02
91	ASV67	Bacteroidetes	Sphingobacteriia	Sphingobacteriales	Chitinophagaceae	Parafilimonas	0.44	0.07
91	ASV21	Bacteroidetes	Sphingobacteriia	Sphingobacteriales	Chitinophagaceae	Parafilimonas	2.31	0.18
91	ASV43	Bacteroidetes	Sphingobacteriia	Sphingobacteriales	Chitinophagaceae	Parafilimonas	1.41	0.12
91	ASV916	Planctomycetes	vadinHA49	unclassified	unclassified	unclassified	0.05	0.01

Table S2. Taxonomy and relative abundance of the transitional bacterial core microbiomes between 0-14, 14-42 and 42-91 days of vermicomposting. Differences between the relative abundance of each ASV at each date are based on paired t-tests (FDR corrected), and significant values are shown in bold.

Gradient	Otu	Phylum	Class	Order	Family	Genus	Mean first date	SE first date	Mean second date	SE second date	*P* -value
0_14	ASV17	Proteobacteria	Gammaproteobacteria	Enterobacteriales	Enterobacteriaceae	unclassified	0.259	1.719	0.054	0.286	**0.021**
0_14	ASV30	Proteobacteria	Alphaproteobacteria	Caulobacterales	Caulobacteraceae	Brevundimonas	0.093	1.407	0.010	0.718	0.139
0_14	ASV7	Bacteroidetes	Cytophagia	Cytophagales	Cytophagaceae	Leadbetterella	0.031	4.990	0.010	1.425	**0.039**
14_42	ASV35	Actinobacteria	Actinobacteria	Micrococcales	Cellulomonadaceae	unclassified	0.196	1.135	0.052	0.146	**0.030**
14_42	ASV119	Firmicutes	Bacilli	Lactobacillales	Leuconostocaceae	Weissella	0.273	0.131	0.054	0.022	0.104
14_42	ASV317	Acidobacteria	Blastocatellia	Blastocatellales	Blastocatellaceae_(Subgroup_4)	Blastocatella	0.053	0.092	0.023	0.012	0.274
14_42	ASV177	Proteobacteria	Betaproteobacteria	Burkholderiales	Alcaligenaceae	Parapusillimonas	0.132	0.130	0.028	0.065	0.984
14_42	ASV31	Proteobacteria	Betaproteobacteria	Burkholderiales	Alcaligenaceae	Achromobacter	0.727	0.858	0.116	0.157	0.698
14_42	ASV22	Proteobacteria	Betaproteobacteria	Burkholderiales	Alcaligenaceae	Achromobacter	1.302	0.891	0.301	0.081	0.342
14_42	ASV87	Proteobacteria	Betaproteobacteria	Burkholderiales	Comamonadaceae	unclassified	0.213	0.405	0.041	0.145	0.342
14_42	ASV51	Proteobacteria	Betaproteobacteria	Burkholderiales	Comamonadaceae	unclassified	0.364	0.918	0.207	0.150	0.206
14_42	ASV54	Proteobacteria	Betaproteobacteria	Burkholderiales	Comamonadaceae	Hydrogenophaga	0.449	0.730	0.062	0.148	0.387
14_42	ASV89	Proteobacteria	Gammaproteobacteria	Pseudomonadales	Pseudomonadaceae	Pseudomonas	0.303	0.239	0.087	0.104	0.703
14_42	ASV74	Proteobacteria	Gammaproteobacteria	Pseudomonadales	Pseudomonadaceae	Pseudomonas	0.477	0.217	0.293	0.013	0.629
14_42	ASV171	Proteobacteria	Gammaproteobacteria	Enterobacteriales	Enterobacteriaceae	unclassified	1.719	0.464	0.286	0.075	0.090
14_42	ASV29	Proteobacteria	Gammaproteobacteria	Xanthomonadales	Xanthomonadaceae	Stenotrophomonas	0.658	1.331	0.200	1.140	0.698
14_42	ASV127	Proteobacteria	Alphaproteobacteria	Caulobacterales	Caulobacteraceae	Brevundimonas	0.241	0.137	0.119	0.045	0.387
14_42	ASV8	Proteobacteria	Alphaproteobacteria	Caulobacterales	Caulobacteraceae	Asticcacaulis	3.440	2.135	0.796	1.378	0.680
14_42	ASV201	Proteobacteria	Alphaproteobacteria	Rhizobiales	Rhizobiaceae	Rhizobium	0.103	0.185	0.045	0.102	0.680
14_42	ASV52	Proteobacteria	Alphaproteobacteria	Rhizobiales	Hyphomicrobiaceae	Devosia	0.442	0.615	0.203	0.132	0.675
14_42	ASV47	Proteobacteria	Alphaproteobacteria	Rhizobiales	Hyphomicrobiaceae	Devosia	0.149	0.752	0.073	0.077	**0.030**
14_42	ASV181	Firmicutes	Clostridia	Clostridiales	Clostridiaceae_1	Clostridium_sensu_stricto_3	0.186	0.088	0.027	0.019	**0.030**
14_42	ASV117	Bacteroidetes	Sphingobacteriia	Sphingobacteriales	Sphingobacteriaceae	unclassified	0.297	0.125	0.113	0.029	0.412
14_42	ASV25	Chlorobi	Chlorobia	Chlorobiales	OPB56	unclassified	0.520	2.314	0.298	0.525	0.175
14_42	ASV122	Bacteroidetes	Sphingobacteriia	Sphingobacteriales	Chitinophagaceae	Flavihumibacter	0.104	0.418	0.024	0.091	0.126
42_91	ASV24	Actinobacteria	Thermoleophilia	Gaiellales	Gaiellaceae	Gaiella	0.644	2.330	0.087	0.284	**0.019**
42_91	ASV64	Actinobacteria	Thermoleophilia	Solirubrobacterales	Elev-16S-1332	unclassified	0.285	0.805	0.045	0.083	**0.019**
42_91	ASV214	Actinobacteria	Thermoleophilia	Solirubrobacterales	Solirubrobacteraceae	Solirubrobacter	0.144	0.176	0.023	0.031	0.371
42_91	ASV37	Actinobacteria	Actinobacteria	Kineosporiales	Kineosporiaceae	unclassified	1.455	0.632	0.250	0.079	0.075
42_91	ASV45	Actinobacteria	Actinobacteria	Micrococcales	Promicromonosporaceae	Cellulosimicrobium	0.941	0.730	0.172	0.091	0.361
42_91	ASV351	Actinobacteria	Actinobacteria	Micrococcales	Cellulomonadaceae	unclassified	1.135	0.607	0.146	0.088	**0.044**
42_91	ASV77	Actinobacteria	Actinobacteria	Corynebacteriales	Mycobacteriaceae	Mycobacterium	0.460	0.477	0.063	0.097	0.889
42_91	ASV62	Chloroflexi	KD4-96	unclassified	unclassified	unclassified	0.186	1.053	0.039	0.228	0.055
42_91	ASV95	Acidobacteria	Solibacteres	Solibacterales	Solibacteraceae_(Subgroup_3)	unclassified	0.323	0.506	0.048	0.057	0.212
42_91	ASV124	Acidobacteria	Solibacteres	Solibacterales	Solibacteraceae_(Subgroup_3)	Bryobacter	0.172	0.357	0.030	0.025	0.075
42_91	ASV118	Gemmatimonadetes	Gemmatimonadetes	Gemmatimonadales	Gemmatimonadaceae	unclassified	0.243	0.357	0.037	0.077	0.415
42_91	ASV251	Verrucomicrobia	Verrucomicrobiae	Verrucomicrobiales	Verrucomicrobiaceae	unclassified	0.137	0.136	0.008	0.021	0.966
42_91	ASV221	Proteobacteria	Betaproteobacteria	Burkholderiales	Alcaligenaceae	Achromobacter	0.891	0.140	0.081	0.014	**0.017**
42_91	ASV112	Proteobacteria	Betaproteobacteria	Burkholderiales	Comamonadaceae	Xenophilus	0.408	0.301	0.083	0.029	0.384
42_91	ASV58	Proteobacteria	Betaproteobacteria	Burkholderiales	Comamonadaceae	unclassified	0.958	0.497	0.231	0.025	0.212
42_91	ASV13	Proteobacteria	Betaproteobacteria	Burkholderiales	Comamonadaceae	unclassified	2.153	2.281	0.296	0.096	0.814
42_91	ASV73	Proteobacteria	Betaproteobacteria	Burkholderiales	Comamonadaceae	unclassified	0.489	0.607	0.090	0.059	0.415
42_91	ASV19	Proteobacteria	Betaproteobacteria	SC-I-84	unclassified	unclassified	0.614	2.976	0.084	0.245	**0.017**
42_91	ASV104	Proteobacteria	Betaproteobacteria	SC-I-84	unclassified	unclassified	0.530	0.276	0.106	0.047	0.169
42_91	ASV98	Proteobacteria	Betaproteobacteria	SC-I-84	unclassified	unclassified	0.214	0.503	0.039	0.045	**0.025**
42_91	ASV156	Proteobacteria	Gammaproteobacteria	Cellvibrionales	Cellvibrionaceae	Cellvibrio	0.199	0.294	0.014	0.039	0.212
42_91	ASV40	Proteobacteria	Gammaproteobacteria	Xanthomonadales	Xanthomonadaceae	Rhodanobacter	0.573	1.347	0.104	0.181	0.076
42_91	ASV71	Proteobacteria	Deltaproteobacteria	Oligoflexales	Oligoflexaceae	Oligoflexus	0.923	0.212	0.097	0.018	**0.017**
42_91	ASV50	Proteobacteria	Deltaproteobacteria	Myxococcales	Sandaracinaceae	unclassified	1.193	0.536	0.215	0.090	0.137
42_91	ASV80	Proteobacteria	Deltaproteobacteria	Myxococcales	Sandaracinaceae	unclassified	0.812	0.207	0.115	0.040	**0.049**
42_91	ASV82	Proteobacteria	Alphaproteobacteria	Caulobacterales	Caulobacteraceae	Phenylobacterium	0.357	0.319	0.071	0.039	0.679
42_91	ASV97	Proteobacteria	Alphaproteobacteria	Caulobacterales	Caulobacteraceae	Asticcacaulis	0.566	0.311	0.116	0.061	0.259
42_91	ASV471	Proteobacteria	Alphaproteobacteria	Rhizobiales	Hyphomicrobiaceae	Devosia	0.752	0.635	0.077	0.086	0.537
42_91	ASV197	Proteobacteria	Alphaproteobacteria	Sphingomonadales	Sphingomonadaceae	Sphingomonas	0.153	0.229	0.020	0.054	0.415
42_91	ASV49	Proteobacteria	Alphaproteobacteria	Sphingomonadales	Sphingomonadaceae	Novosphingobium	1.005	0.582	0.128	0.053	0.075
42_91	ASV39	Proteobacteria	Alphaproteobacteria	Sphingomonadales	Sphingomonadaceae	Novosphingobium	0.656	0.998	0.134	0.101	0.114
42_91	ASV132	Proteobacteria	Alphaproteobacteria	Sphingomonadales	Erythrobacteraceae	Altererythrobacter	0.263	0.346	0.078	0.058	0.291
42_91	ASV110	Proteobacteria	Alphaproteobacteria	Rhodobacterales	Rhodobacteraceae	unclassified	0.295	0.380	0.059	0.072	0.419
42_91	ASV102	Proteobacteria	Alphaproteobacteria	Rhodobacterales	Rhodobacteraceae	unclassified	0.350	0.344	0.046	0.099	0.966
42_91	ASV55	Proteobacteria	Alphaproteobacteria	Rhodobacterales	Rhodobacteraceae	unclassified	0.537	0.840	0.089	0.218	0.361
42_91	ASV41	Proteobacteria	Alphaproteobacteria	unclassified	unclassified	unclassified	0.570	1.130	0.127	0.202	0.068
42_91	ASV193	Proteobacteria	Alphaproteobacteria	unclassified	unclassified	unclassified	0.243	0.125	0.038	0.045	0.150
42_91	ASV274	Firmicutes	Clostridia	Clostridiales	Lachnospiraceae	Mobilitalea	0.096	0.079	0.038	0.014	0.679
42_91	ASV252	Chlorobi	Chlorobia	Chlorobiales	OPB56	unclassified	2.314	0.141	0.525	0.037	0.055
42_91	ASV3	Bacteroidetes	Sphingobacteriia	Sphingobacteriales	Chitinophagaceae	unclassified	2.217	14.447	0.394	1.238	**0.017**
42_91	ASV12	Bacteroidetes	Sphingobacteriia	Sphingobacteriales	Chitinophagaceae	unclassified	1.686	3.071	0.284	0.256	0.099
42_91	ASV69	Bacteroidetes	Sphingobacteriia	Sphingobacteriales	Chitinophagaceae	Filimonas	0.934	0.237	0.130	0.052	0.055
42_91	ASV67	Bacteroidetes	Sphingobacteriia	Sphingobacteriales	Chitinophagaceae	Parafilimonas	0.743	0.438	0.102	0.067	0.188
42_91	ASV21	Bacteroidetes	Sphingobacteriia	Sphingobacteriales	Chitinophagaceae	Parafilimonas	1.018	2.310	0.156	0.180	**0.049**

Table S3. Relative abundances of microbial genes implied in lignocellulose degradation during decomposition of the Scotch broom. Mean±SE.

	KEGG gene description [EC number]	0 days	14 days	42 days	91 days
Lignin	Glycolate oxidase [EC:1.1.3.15]	0.20±0.04a	4.64±0.46b	7.81±0.28c	7.36±0.35c
	Catalase [EC:1.11.1.6]	7.58±0.05a	4.53±.18b	4.05±0.13c	4.07±0.05c
	Vanillate monooxygenase [EC:1.14.13.82]	9.21±0.10a	3.60±0.17b	4.47±0.27c	4.20±0.06d
	Catalase/peroxidase [EC:1.11.1.6 1.11.1.7]	5.37±0.04a	4.21±00.15b	5.17±0.13a	5.13±0.03a
	Glutathione peroxidase [EC:1.11.1.9]	6.46±0.05a	4.91±0.09b	4.46±0.11c	4.41±0.07d
	Cytochrome c peroxidase [EC:1.11.1.5]	0.21±0.02a	4.68±0.20b	5.98±0.28c	6.81±0.49d
	Chloride peroxidase [EC:1.11.1.10]	6.38±0.08a	4.76±0.15b	4.78±0.12b	4.58±0.16c
	Thiol peroxidase. atypical 2-Cys peroxiredoxin [EC:1.11.1.15]	10.57±0.02a	5.01±0.23b	2.26±0.16c	2.25±0.05c
	Peroxiredoxin (alkyl hydroperoxide reductase subunit C) [EC:1.11.1.15]	6.27±0.02a	5.29±0.12b	4.10±0.09c	4.35±0.06d
(Hemi)cellulose	Alpha-amylase [EC:3.2.1.1]	13.67±0.10a	3.57±0.32b	1.58±0.07c	1.18±0.04c
	Alpha-galactosidase [EC:3.2.1.22]	4.05±0.05a	6.24±0.23b	4.69±0.24c	5.02±0.08c
	Alpha-L-fucosidase [EC:3.2.1.51]	0.06±0.01a	7.20±0.41bd	5.42±0.23c	7.31±0.53d
	Alpha-mannosidase [EC:3.2.1.24]	2.19±0.11a	10.26±0.77b	3.57±0.29ac	3.56±0.36c
	Alpha-N-arabinofuranosidase [EC:3.2.1.55]	0.54±0.02a	8.86±0.57b	6.05±0.44c	5.96±00.18d
	Arabinogalactan endo-1.4-beta-galactosidase [EC:3.2.1.89]	10.77±0.15a	5.02±0.48b	2.79±0.28c	2.57±0.03d
	Beta-galactosidase [EC:3.2.1.23]	3.65±0.01a	8.86±0.28b	3.81±0.45a	4.00±0.23a
	Beta-glucuronidase [EC:3.2.1.31]	0.05±0.01a	6.26±0.71b	6.97±1.08b	7.03±0.21b
	Beta-mannosidase [EC:3.2.1.25]	0.08±0.01a	11.54±0.76b	5.27±0.68c	4.95±0.15d
	Carboxylesterase [EC:3.1.1.1]	1.51±0.47a	9.37±0.91b	5.39±0.41c	5.08±0.05d
	Endo-1.4-beta-xylanase [EC:3.2.1.8]	0.07±0.01a	7.12±0.31b	6.78±0.25b	6.75±0.09c

	Evolved beta-galactosidase subunit alpha [EC:3.2.1.23]	19.36±0.3a	0.28±0.04b	0.21±0.02b	0.19±0.01b
	Levanase [EC:3.2.1.65]	1.63±0.09a	8.74±0.84b	3.80±0.21c	4.49±0.47d
	Lysophospholipase [EC:3.1.1.5]	11.2±0.05a	2.95±0.18bc	3.27±0.24b	3.12±0.14c
	Mannan endo-1.4-beta-mannosidase [EC:3.2.1.78]	0.02±0.01a	12.91±1.35b	4.38±0.41c	4.08±0.12c
	Xylan 1,4-beta-xylosidase [EC:3.2.1.37]	1.08±0.05a	6.17±0.74b	7.07±0.80b	7.18±0.31b
	Endoglucanase [EC:3.2.1.4]	4.47±0.03a	3.37±0.26b	5.42±0.24c	5.63±0.11d
Cellobiose	6-phospho-beta-glucosidase [EC:3.2.1.86]	18.05±0.2a	1.24±0.16b	0.37±0.03c	0.34±0.03c
	Alpha-glucosidase [EC:3.2.1.20]	4.20±0.05a	7.29±0.38b	4.27±0.20a	4.24±0.04a
	Beta-glucosidase [EC:3.2.1.21]	9.54±0.11a	2.43±0.23b	4.28±0.13c	4.08±0.17d
	Glucan endo-1.3-beta-D-glucosidase [EC:3.2.1.39]	0.01±0.01a	3.45±0.55b	9.66±0.40c	6.89±0.53d
	Oligo-1.6-glucosidase [EC:3.2.1.10]	12.88±0.2a	1.64±0.20b	2.98±0.26c	2.50±0.10c
Cello-oligosaccharides	Beta-fructofuranosidase [EC:3.2.1.26]	13.98±0.1a	3.15±0.34b	1.37±0.08c	1.50±0.10c
	Glyceraldehyde 3-phosphate dehydrogenase [EC:1.2.1.12]	5.88±0.03a	4.72±0.05b	4.56±0.03c	4.84±0.05b
	Alpha,alpha-trehalase [EC:3.2.1.28]	11.1±0.07a	3.70±0.54b	2.16±0.18c	3.02±0.27bc
	Trehalose-6-phosphate hydrolase [EC:3.2.1.93]	18.8±0.23a	0.84±0.11b	0.20±0.04c	0.17±0.01c
	Maltose-6CE-phosphate glucosidase [EC:3.2.1.122]	12.98±0.62a	4.75±0.63b	1.04±0.14c	1.23±0.13c
	Alpha-L-rhamnosidase [EC:3.2.1.40]	4.41±0.07a	6.94±0.47b	3.76±0.16ac	4.89±0.30ad
	Maltooligosyltrehalose trehalohydrolase [EC:3.2.1.141]	0.45±0.14a	4.16±0.47b	7.87±0.38c	7.52±0.35c
	PTS system. cellobiose-specific IIA component [EC:2.7.1.69]	17.61±0.14a	1.58±0.22b	0.35±0.04c	0.47±0.04c
	PTS system. cellobiose-specific IIB component [EC:2.7.1.69]	12.80±1.35a	5.41±2.04b	0.51±0.03c	1.28±0.17c
	PTS system. cellobiose-specific IIC component	17.96±0.17a	1.42±0.21b	0.30±0.04c	0.32±0.03c
